# Improved lipid production and component of mycosporine-like amino acids by co-overexpression of *amt1* and *aroB* genes in *Synechocystis* sp. PCC6803

**DOI:** 10.1038/s41598-023-46290-x

**Published:** 2023-11-09

**Authors:** Kamonchanock Eungrasamee, Peter Lindblad, Saowarath Jantaro

**Affiliations:** 1 Laboratory of Cyanobacterial Biotechnology, Department of Biochemistry, Faculty of Science, Chulalongkorn University, Bangkok, 10330 Thailand; 2 Microbial Chemistry, Department of Chemistry – Ångström, Uppsala University, Box 523, 75120 Uppsala, Sweden

**Keywords:** Biochemistry, Biotechnology, Microbiology

## Abstract

Implementing homologous overexpression of the *amt1* (A) and *aroB* (B) genes involved in ammonium transporter and the synthesis of mycosporine-like amino acids (MAAs) and aromatic amino acids, respectively, we created three engineered *Synechocystis* sp. PCC6803 strains, including Ox-A, Ox-B, and Ox-AB, to study the utilization of carbon and nitrogen in cyanobacteria for the production of valuable products. With respect to *amt1* overexpression, the Ox-A and Ox-AB strains had a greater growth rate under (NH_4_)_2_SO_4_ supplemented condition. Both the higher level of intracellular accumulation of lipids in Ox-A and Ox-AB as well as the increased secretion of free fatty acids from the Ox-A strain were impacted by the late-log phase of cell growth. It is noteworthy that among all strains, the Ox-B strain undoubtedly spotted a substantial accumulation of glycogen as a consequence of *aroB* overexpression. Additionally, the ammonium condition drove the potent antioxidant activity in Ox strains with a late-log phase, particularly in the Ox-B and Ox-AB strains. This was probably related to the altered MAA component inside the cells. The higher proportion of P4-fraction was induced by the ammonium condition in both Ox-B and Ox-AB, while the noted increase of the P1 component was found in the Ox-A strain.

## Introduction

Cyanobacteria are Gram-negative bacteria possessing photosynthesis, which makes them require nitrogen and carbon sources from sunlight, H_2_O, carbon dioxide, and some minimal minerals, and then convert them to valuable products, such as chemicals and bioenergy compounds^1–4^. Carbon fixation is an efficient reaction in the Calvin-Benson-Bassham (CBB) cycle that takes place in the carboxysome, while cyanobacteria commonly utilize various kinds of nitrogen sources, such as ammonium, nitrate, nitrite, urea, atmospheric N_2_, and arginine, mostly through the systems of diffusion and ATP-binding cassettes^5,6^. Cyanobacterial nitrate assimilation is controlled by the altered 2-oxoglutarate signal in line with the altered cellular C/N balance and hindered by the presence of ammonium in the growth medium^7–10^. In the cyanobacterium *Synechocystis* sp. PCC 6803, genetically overexpressed *RuBisCO* genes in the CBB cycle certainly enhanced the carbon pool for increased lipids and polyhydroxybutyrate (PHB) bioplastic synthesis, particularly under nitrogen and phosphorus deprivation^4,11^. While nitrogen is a crucial nutrient source for the biosynthesis of organic N-containing compounds and basic biomolecules such as amino acids, proteins, nucleic acids, and carbohydrates, it also has a tight connection with nitrogen transport and assimilation^12,13^. There are four proteins found for the ABC-type of nitrate transporter (NRT), including NrtA, NrtB, NrtC, and NrtD in cyanobacteria^14^, whereas ammonium is transported via the ammonium/methylammonium permeases family encoded by *amt1* (*sll0108*), *amt2 (sll1017*), and *amt3* (*sll0537*) genes, and amt1, being mainly responsible for ammonium uptake, has the highest affinity activity by about 95% of permease activity^15^.

The outlined connection between the metabolism of carbon and nitrogen in cyanobacteria is a well-known process^1,16,17^. Through the sequential action of two enzymes, including glutamine synthetase (GS) and glutamate synthase (GOGAT), in the glutamate/glutamine cycle, ammonium is directly transferred from the media via the Amt transporter and integrated into carbon skeletons^10,16^. The amidation of glutamate to glutamine is catalyzed by the GS enzyme, and the GOGAT enzyme catalyzes the reductive transfer of the amide group from glutamine to 2-oxoglutarate (2-OG)^18^. Despite the fact that cyanobacteria lack 2-oxoglutarate dehydrogenase, it was found that 2-oxoglutarate decarboxylase (OGDC) and succinic semialdehyde dehydrogenase (SSA-DH) work together to fill the gap between 2-OG and succinate in the TCA cycle^19^. The alternative route to succinate is the γ-aminobutyrate (GABA) shunt from glutamate which is composed of glutamate decarboxylase (GDC), GABA aminotransferase (GABA-AT), and SSA-DH^20,21^. A malic enzyme (ME) converts malate to pyruvate, which releases CO_2_, and proceeds to flow in a number of other routes, including the formation of acetyl-CoA, phosphoenolphyruvate (PEP), carotenoids, and isoprene molecules. In one hand, main acetyl-CoA precursor could flow to many main pathways, including TCA cycle, fatty acid and lipid syntheses (FAS II), and polyhydroxybutyrate (PHB) production^22–26^. In cyanobacteria, membrane lipids can be hydrolyzed by lipase A enzyme, which is encoded by *lipA* gene. The thereby produced free fatty acids (FFAs) can then be recycled into fatty acyl-ACP by the acyl-ACP synthetase, encoded by *aas* gene^1,2,27,28^, or they can be secreted outside of the cell as extracellular FFAs. On the other direction, pyruvate can be converted to phosphoenolpyruvate (PEP) by phosphoenolpyruvate synthase (PPS). To synthesize aromatic amino acids and mycosporine-like amino acids (MAAs), PEP combines with erythrose-4-phosphate (E4P) to generate 3-deoxy-D-arabino-heptulosonate-7-phosphate (DAHP) which can be converted to 3-dehydroquinate (DHQ) by the 3-dehydroquinate synthase enzyme, encoded by *aroB* (*slr2130*)*.* The DHQ is an important precursor for aromatic amino acid syntheses, and 4-deoxygadusol (4-DG) which is the substrate for the syntheses of MAA and MAA derivatives^29^. To date, over 40 different derivative MAAs and MAAs, such as shinorine, porphyra-334, palythine, and mycosporine-2-glycine, have been identified^30,31^ and are well-known as photoprotectants or neutrally cellular sunscreens due to their chemical structures^32^. MAAs might be considered as an intracellular nitrogen reserve, although the processes regulating their breakdown are currently unclear^33,34^.

In this study, we constructed three mutant strains, including *Synechocystis* sp. PCC 6803 overexpressing the *amt1* (or *sll0108*) gene encoding NH_4_^+^ permease involved in ammonium transporter (Ox-A strain), *Synechocystis* sp. PCC 6803 overexpressing the *aroB* (or *slr2130*) gene encoding 3-dehydroquinate (DHQ) synthase involved in mycosporine-like amino acid (MAA) and aromatic amino acid syntheses (Ox-B), and a double overexpression of the Ox-AB strain. With a system of (NH_4_)_2_SO_4_ supplementation, we discovered a substantial flow between nitrogen and carbon metabolism in these modified strains. All Ox strains had elevated lipid levels, and various components of MAAs were also present.

## Results

### Engineered *Synechocystis* sp. PCC6803 strains

**Figure 1:**
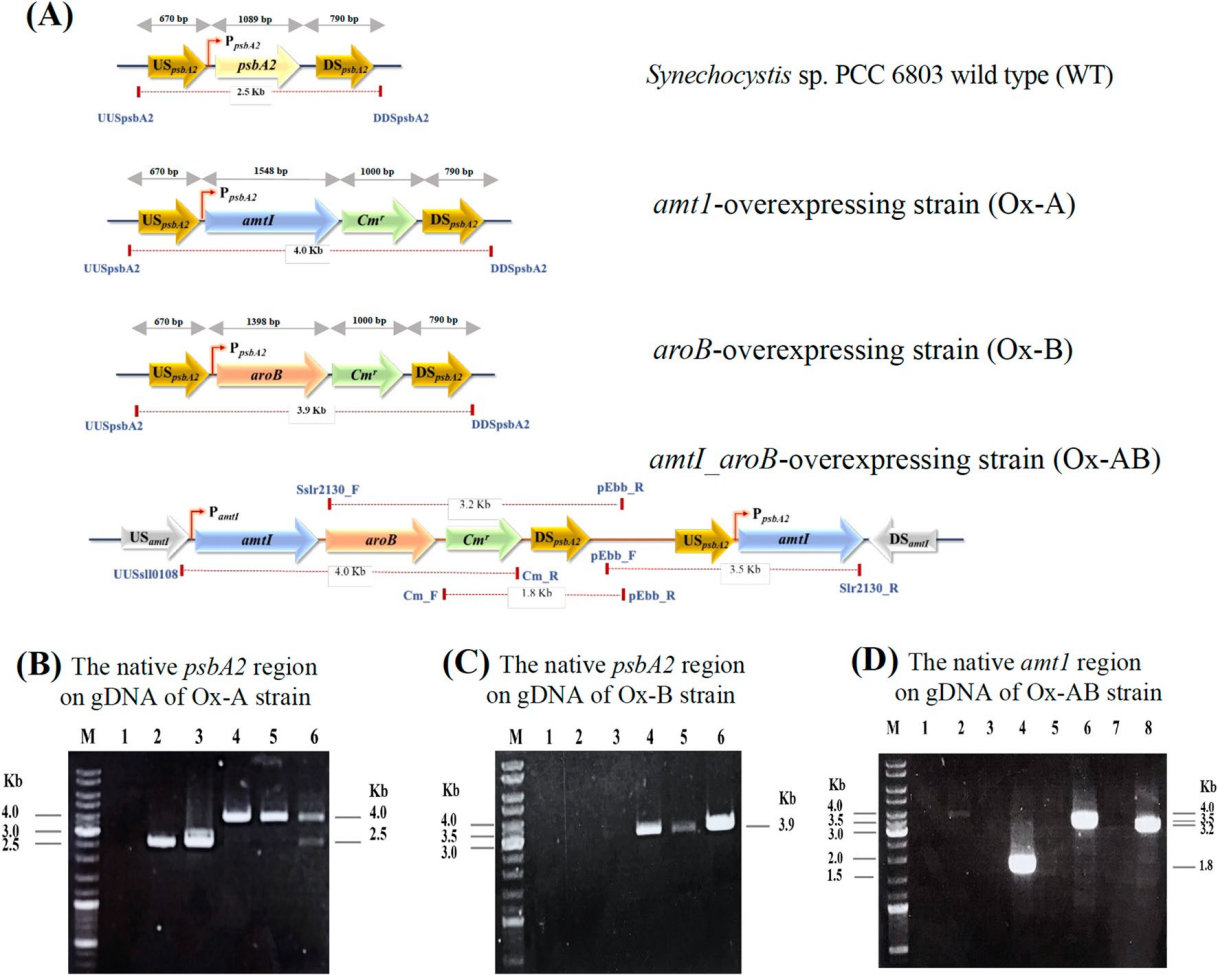
Genomic maps of the engineered *Synechocystis* strains, including Ox-A (**A**, **B**), Ox-B (**A**, **C**), and Ox-AB (**A**, **D**), respectively. The specific pairs of primers (Supplementary Information Table S1) were used to confirm the complete integration of all transformants by PCR analysis. For the double homologous recombination (**A**), the *amt1* or *aroB* gene recombination occurred between the conserved sequences of *psbA2* gene in *Synechocystis* sp. PCC 6803 wild type (WT). For Ox-A strain (**B**), PCR products using UUSpsbA2 and DDSpsbA2 primers, Lane M: GeneRuler DNA ladder (Fermentas Life Sciences, MD, USA); Lane 1: negative control using WT as template, Lanes 2–6: Clones No. 1-5. Only positive clones no. 3 and 4 in respective Lanes 4 and 5, were obtained. For Ox-B strain (**C**), PCR products using UUSpsbA2 and DDSpsbA2 primers, Lane M: GeneRuler DNA ladder, Lane 1: negative control using WT as template; Lanes 2–6: Clones No. 1-5. Only positive Clones No. 3-5 in respective Lanes 4–6, were obtained. For Ox-AB strain (**D**), the single homologous recombination was confirmed by PCR using various pairs of primers (Supplementary Information Table S1), Lane M: GeneRuler DNA ladder; Lanes 1 and 2: negative control using WT as template and a transformant, respectively, with UUSll0108_F and Cm_R primers; Lanes 3 and 4: negative control using WT as template and a transformant, respectively, with Cm_F and pEbb_R primers, Lanes 5 and 6: negative control using WT as template and a transformant, respectively, with pEbb_F and Slr2130_R primers, Lanes 7 and 8: negative control using WT as template and a transformant, respectively, with Slr2130_F and pEbb_R primers.

**Figure 2:**
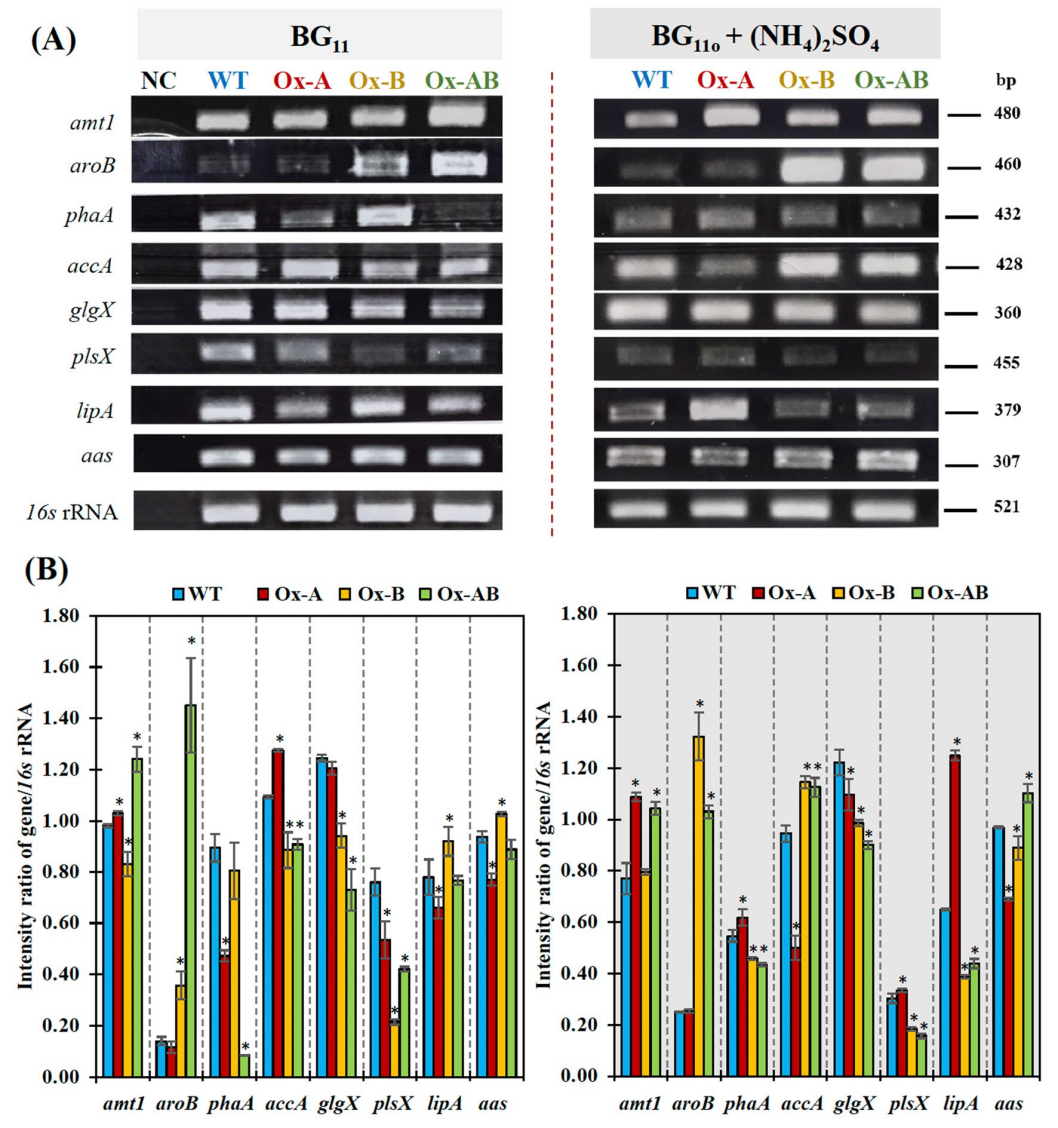
The transcript levels (**A**) and relative transcript intensity ratios (**B**) of the *amt1, aroB, phaA, accA*, *glgX, plsX, lipA, aas,* and *16s* rRNA performed by RT-PCR in *Synechocystis* WT, Ox-A, Ox-B, and Ox-AB strains. Cells were grown in normal BG_11_ and BG_110_ + (NH_4_)_2_SO_4_ media and analyzed at day 10 of cultivation (late-log phase). In (**B**), the error bars represent standard deviations of means (mean ± S.D., n = 3). The statistical difference of the results between the values of WT and engineered strain is represented by an asterisk, *P < 0.05. All cropped gels were taken from the original images of RT-PCR products on agarose gels as shown in Supplementary Information Figures S1 and S2.

Three constructed *Synechocystis* sp. PCC6803 strains, including Ox-A, Ox-B, and Ox-AB (Table 1 and Fig. 1) were obtained by single or double homologous recombination. First, the recombinant plasmids (Table 1), including pECm_*amt1*, pECm_*aroB*, and pECm_*amt1/aroB*, were generated by ligating each amplified gene fragment with pEERM vector. For single and double recombinant plasmids, native *amt1*, *aroB*, and *amt1_aroB* gene fragments were separately ligated between flanking regions of the *psbA2* gene of the pEERM vector and the upstream region of *Cm*^*r*^ cassette (Table 1). For PCR analysis using UUSpsbA2 and DDSpsbA2 as a pair of primers (Supplementary Information Table S1), the positive colonies of Ox-A were clones no. 3 and no. 4 as shown by the expected size of about 4.0 Kb in Lanes 4 and 5, respectively (Fig. 1B). For Ox-B, the PCR product using UUSpsbA2 and DDSpsbA2 primers was found in Clones No. 3-5, as shown in Lanes 4–6 with the fragment size of about 3.9 Kb (Fig. 1C). Those two single gene overexpressing strains were ultimately confirmed by double homologous recombination as expected. For a double overexpression of *amt1* and *aroB* genes in *Synechocystis* WT (Ox-AB), only one positive clone was obtained by single recombination. Its PCR amplifications with various specific pairs of primers, including UUSsll0108/Cm_R, Cm_F/pEbb_R, pEbb_F/Slr2130_R, and slr2130_F/pEbb_R (Supplementary Information Table S1), with the expected sizes of about 4.0, 1.8, 3.5, and 3.2 Kb, respectively, were correctly confirmed in Lanes 2, 4, 6, and 8, respectively, compared with the negative control of each specific pair of primers in Lanes 1, 3, 5, and 7, respectively (Fig. 1D). The higher transcript levels of gene overexpression, including *amt1* of Ox-A, *aroB* of Ox-B, and both of them in Ox-AB, were verified by RT-PCR (Fig. 2A).

**Table 1:** Strains and plasmids used in this study.

Name	Relevant genotype	Reference
Cyanobacterial strains		
* Synechocystis* sp. PCC 6803	Wild type	Pasteur culture collection
Ox-A	*amt1, cm*^*r*^ integrated at the native *psbA2* gene in *Synechocystis* WT genome	This study
Ox-B	*aroB, cm*^*r*^ integrated at the native *psbA2* gene in *Synechocystis* WT genome	This study
Ox-AB	*amt1,aroB, cm*^*r*^ integrated at the native *amt1* gene in *Synechocystis* WT genome	This study
Plasmids		
pEERM	P_psbA2_–*cm*^*r*^ ; plasmid containing c*m*^*r*^ between the flanking region of upstream and downstream *psbA2* sequences	This study
pECm_* amt1*	P_psbA2_–*sll0108-cm*^*r*^ ; plasmid containing *sll0108* and c*m*^*r*^ between the flanking region of upstream and downstream *psbA2* sequences	This study
pECm_*aroB*	P_psbA2_–*slr2130-cm*^*r*^ ; plasmid containing *slr2130* and c*m*^*r*^ between the flanking region of upstream and downstream *psbA2* sequences	This study
pECm_*amt1/aroB*	P_psbA2_–*sll0108-slr2130-cm*^*r*^ ; plasmid containing *sll0108, slr2130* and c*m*^*r*^ between the flanking region of upstream and downstream *psbA2* sequences	This study

*P*_*psbA2*_ strong *psbA2* promoter, *cm*^*r*^ chloramphenicol resistance cassette.

### Growth, intracellular pigments, and oxygen evolution rate of engineered strains

**Figure 3:**
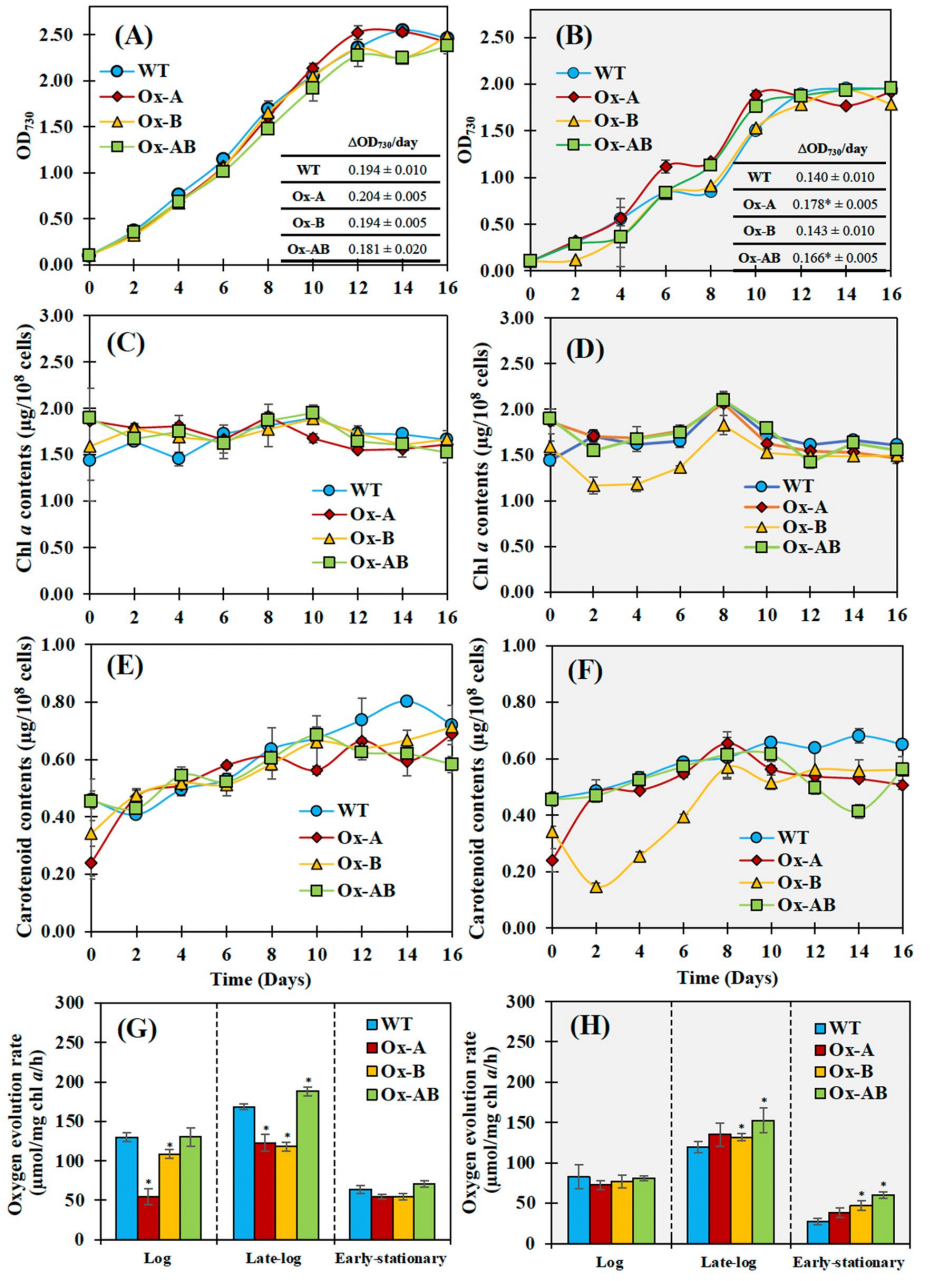
Growth (**A**, **B**), chlorophyll *a* (**C**, **D**) and carotenoid (**E**, **F**) contents, and oxygen evolution rates (**G**, **H**) of *Synechocystis* WT, Ox-A, Ox-B, and Ox-AB strains cultured under normal BG_11_ medium (while background; **A**, **C**, **F**, **G**) and BG_110_ medium with (NH_4_)_2_SO_4_ supplementation (BG_110_ + (NH_4_)_2_SO_4_, gray background; **B**, **D**, **F**, **H**) during 16 day of cultivation. The oxygen evolution rates (**E**, **F**) of all strains were determined from cell culture growing at the log (day 6), late-log (day 10), and early-stationary (day 12) phases. The error bars represent standard deviations of means (mean ± S.D., n = 3) with the statistical difference of the results between WT and engineered strain represented by an asterisk, *P < 0.05.

**Figure 4:**
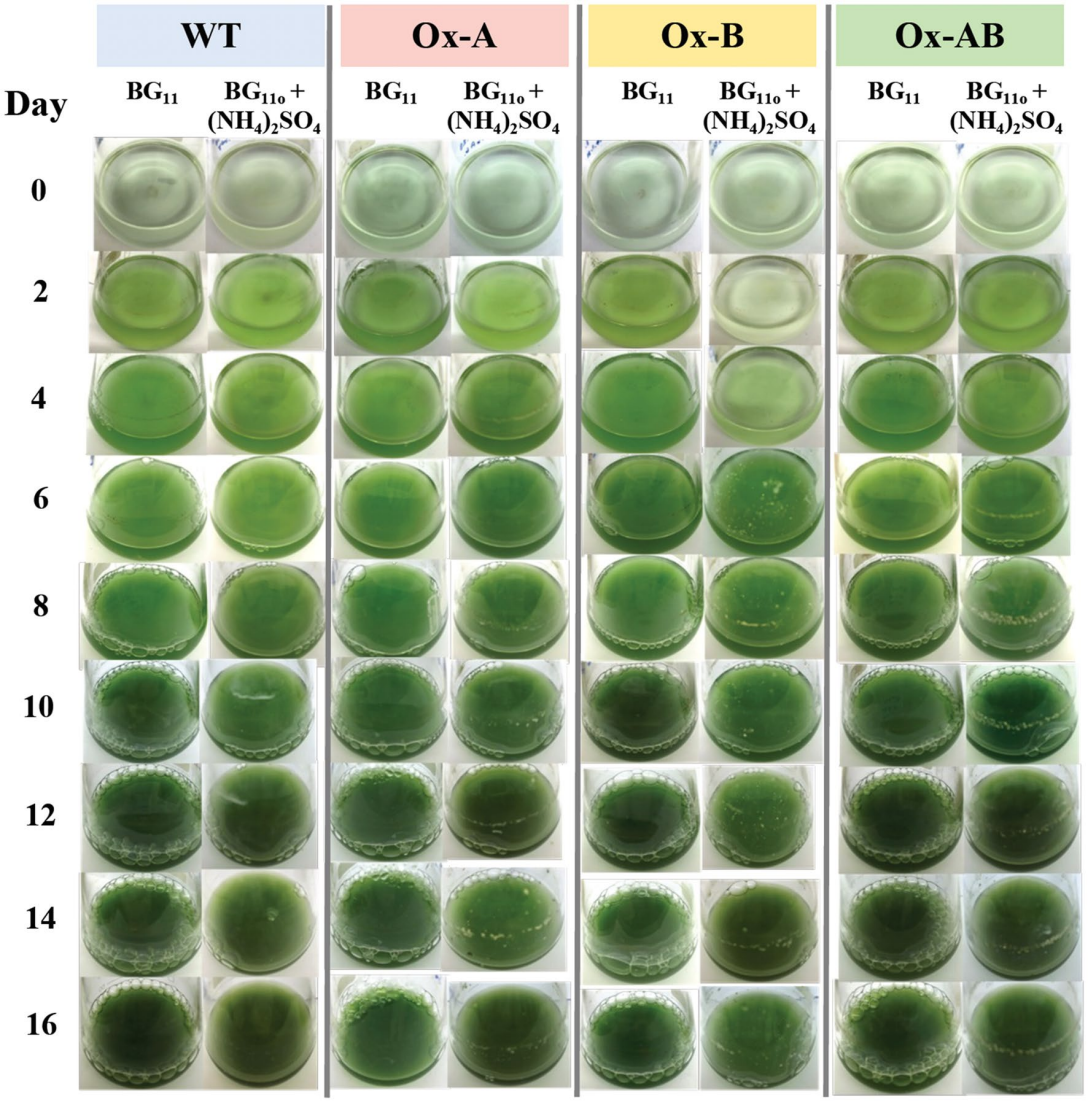
Images of cell cultured flasks of *Synechocystis* WT, Ox-A, Ox-B, and Ox-AB strains, grown in normal BG_11_ and BG_110_ + (NH_4_)_2_SO_4_ media during 16 days of cultivation.

All strains were cultured for 16 days in both regular BG_11_ medium and with 8.8 mM (NH_4_)_2_SO_4_ supplementation in BG_11_ medium without NaNO_3_ (BG_110_ + (NH_4_)_2_SO_4_) (Figs. 3 and 4). It is important to note that the growth of all strains in BG_110_ + (NH_4_)_2_SO_4_ medium was lower than it was in normal BG_11_. However, the *amt1*-overexpressing strain (Ox-A) showed a higher growth rate than other strains under both growth conditions (Fig. 3A,B). As expected, Ox-A and Ox-AB strains with overexpressing *amt1* gene in ammonium transporter had higher growth than WT and Ox-B strains under (NH_4_)_2_SO_4_-supplemented condition, as well as higher growth rates (Fig. 3B). These were in line with the first four days of cultivation, when cell cultured flasks of the Ox-A and Ox-AB strains contained deeper green than those of the WT and Ox-B strains (Fig. 4). On the other hand, the constant accumulation of chlorophyll *a* contents occurred in all strains under both growth conditions, except for the Ox-B under the (NH_4_)_2_SO_4_-supplemented condition, which had the lowest amounts among all strains with light green culture during the first four days of cultivation (Figs. 3C,D, and 4). It was found that all strains noted an enhanced accumulation of carotenoids during the duration of 16 days of growth in normal BG_11_ medium, in particular the WT strain (Fig. 3E). In exception, the Ox-B strain also contained the lowest amount of carotenoids under (NH_4_)_2_SO_4_-supplemented condition among all strains during the first 8 days of growth (Fig. 3F). Additionally, the oxygen evolution rates of all strains were increased during late-log phase of growth, and subsequently decreased during early-stationary phase (Fig. 3G,H). It is worth noting that the Ox-A and Ox-B strains possessed a lower oxygen evolution rate than WT, while Ox-AB has a higher oxygen evolution rate under normal BG_11_ condition regarding its less chlorophyll *a* content (Fig. 3G). The reduction in oxygen evolution rate of the WT and Ox-AB strains was influenced by the BG_110_ + (NH_4_)_2_SO_4_ condition (Fig. 3H).

### Productions of intracellular lipids, extracellular free fatty acids (FFAs), glycogen, and PHB under ammonium sulfate supplementation

**Figure 5:**
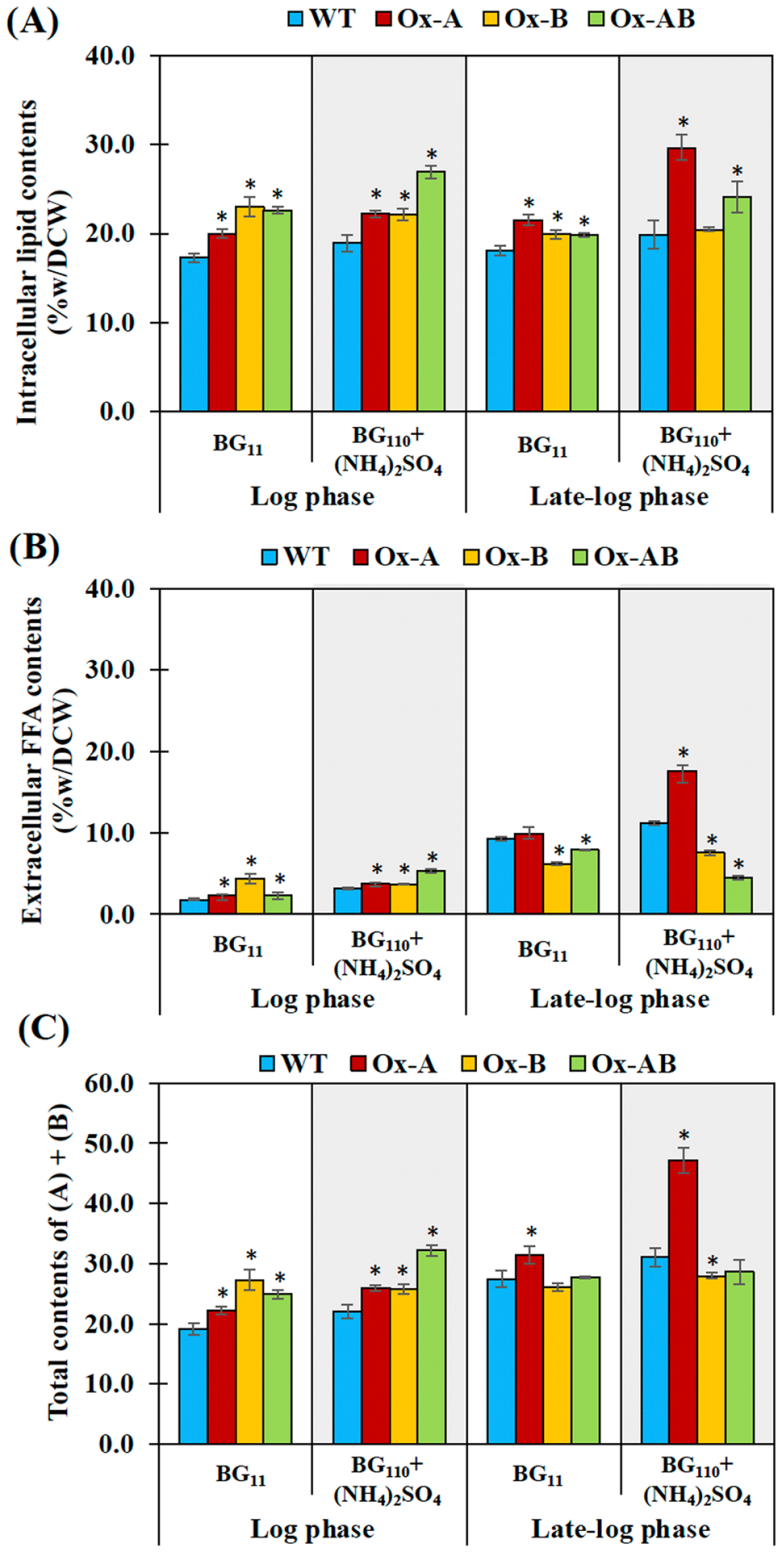
Contents (%w/DCW) of intracellular lipids (**A**), extracellular free fatty acids (FFAs) (**B**), and total contents of intracellular lipids and extracellular FFAs (**C**) in *Synechocystis* WT, Ox-A, Ox-B, and Ox-AB strains. Cells were grown in normal BG_11_ (white background) and BG_110_ + (NH_4_)_2_SO_4_ (gray background) media and analyzed at day 6 and 10 of cultivation represented cells at log phase and late-log phases of cell growth, respectively. The error bars represent standard deviations of means (mean ± S.D., n = 3). The statistical difference of the results between WT and engineered strain was represented by an asterisk, *P < 0.05.

We found increased levels of intracellular lipids in Ox strains, especially Ox-A and Ox-AB strains, during log and late-log phases of growth (Fig. 5A). Under (NH_4_)_2_SO_4_-supplemented condition, the Ox-AB strain accumulated the highest level of intracellular lipids (26.9%w/DCW) during the log phase, while Ox-A had the highest content of intracellular lipids (29.6%w/DCW) during late-log phase of growth. Late-log phase of growth was primarily prominent for higher secretion of FFAs than log phase, while (NH_4_)_2_SO_4_ supplementation synergistically increased the secretion of FFAs, in particular the Ox-A strain (17.5%w/DCW) (Fig. 5B). The Ox strains in this study had higher total amounts of intracellular lipids and extracellular FFAs at the log phase of cell growth, as presented in Fig. 5C, while at the late-log phase, only Ox-A strain contained the highest total amount of them at about 47.2%w/DCW (Fig. 5C).

**Figure 6:**
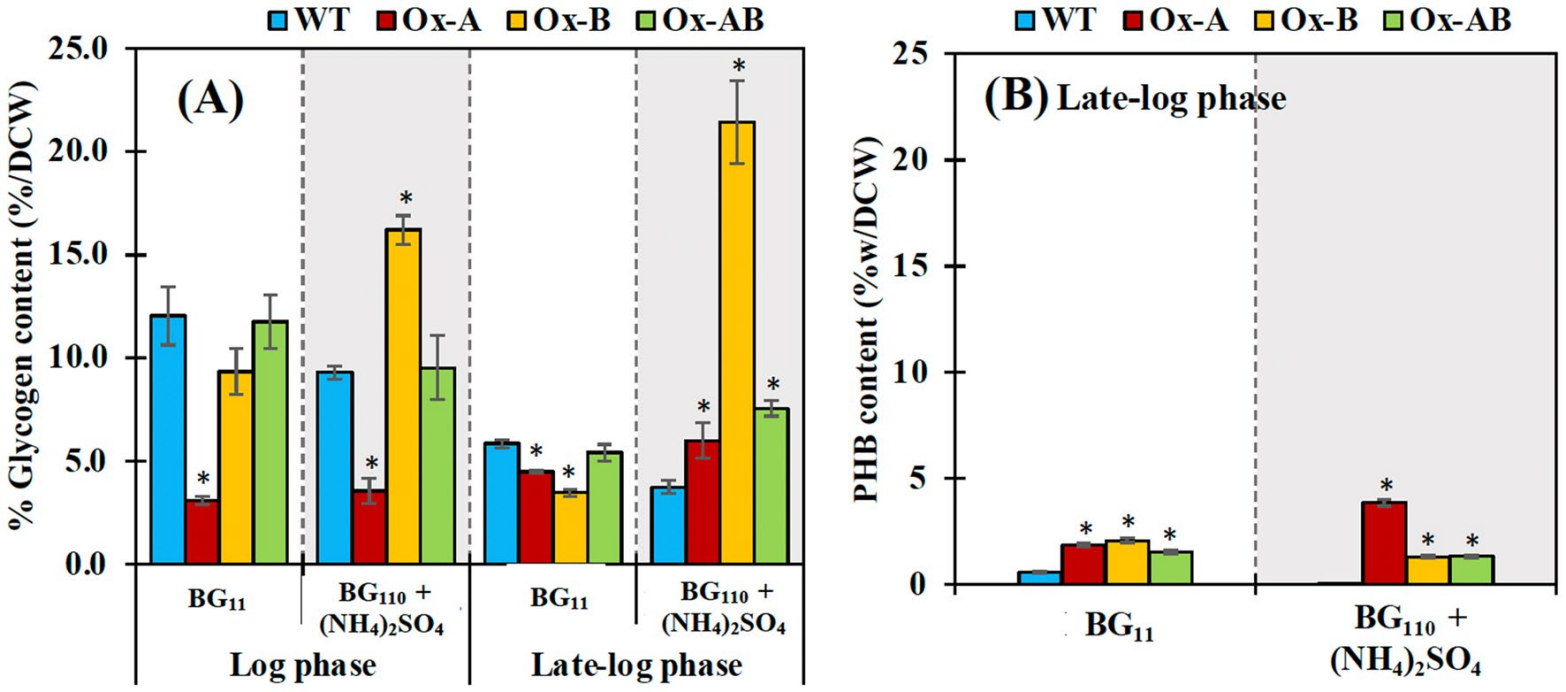
The glycogen (**A**) and PHB (**B**) contents of WT, Ox-A, Ox-B, and Ox-AB strains. Cells were grown in normal BG_11_ and BG_110_ + (NH_4_)_2_SO_4_ media. The error bars represent standard deviations of means (mean ± S.D., n = 3). The statistical difference of the results between WT and engineered strain was represented by an asterisk, *P < 0.05.

**Figure 7:**
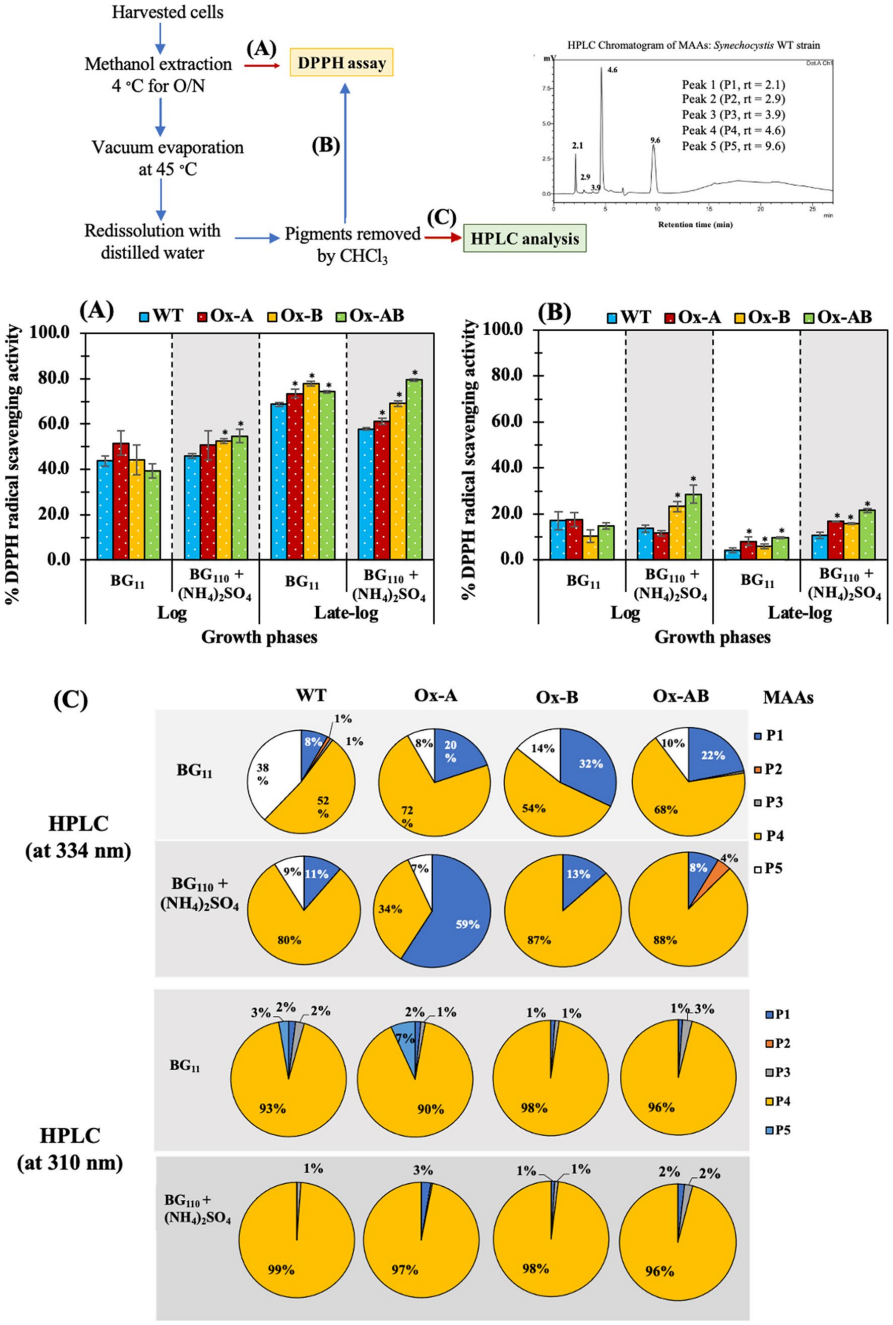
DPPH radical scavenging activity (**A**, **B**) and compositions of mycosporine-like amino acids (**C**) of *Synechocystis* WT, Ox-A, Ox-B, and Ox-AB strains. Cells were cultured in normal BG_11_ and BG_110_ + (NH_4_)_2_SO_4_ media and analyzed at log (day 6) and late-log (day 10) phases of cell growth. The DPPH radical scavenging activities were determined in methanol extract (**A**), and pigment-free methanol extract (**B**). The error bars represent standard deviations of means (mean ± S.D., n = 3). The statistical difference of the results between WT and engineered strain was represented by an asterisk, *P < 0.05. (**C**) The mycosporine-like amino acid (MAA) compositions in methanol extract from all strains were detected by HPLC at 334 and 310 nm, including Peak 1 (P1, rt = 2.3), Peak 2 (P2, rt = 2.9), Peak 3 (P3, rt = 3.9), Peak 4 (P4, rt = 4.6), and Peak 5 (P5, rt = 9.5).

The other linkage metabolites related to carbon storage in cyanobacteria, including glycogen and PHB accumulation, were also determined (Fig. 6). The least glycogen content was found in the Ox-A strain at the log phase of cell growth under both growth conditions, while the higher accumulation of glycogen was induced by (NH_4_)_2_SO_4_ in the Ox-B strain at approximately 16.2 and 21.4% w/DCW at the log and late-log phases, respectively (Fig. 6A). It is important to point out that the growth phase had a relationship with the glycogen accumulation, as clearly indicated by the reduced glycogen content noticed in the late-log phase. Moreover, the (NH_4_)_2_SO_4_ supplementation could decrease glycogen accumulation in WT strain at both log and late-log phases of growth (Fig. 6A). On the other hand, polyhydroxybutyrate (PHB) accumulation, another carbon storage in cyanobacteria, was trivially induced in Ox strains that were grown in normal BG_11_ and BG_110_ + (NH_4_)_2_SO_4_ media (Fig. 7B). However, (NH_4_)_2_SO_4_ treatment could slightly induce PHB production in the Ox-A strain (3.85%w/DCW), but almost abolished PHB accumulation in the WT strain.

Moreover, the transcript levels of genes including, *amt1*, *aroB, phaA, accA, glgX, plsX, lipA, aas, 16s* rRNA in all strains grown at late-log phase of growth under both conditions were shown in Fig. 2. The transcript amount of the *amt1* gene, encoding the AMT transporter, was normally expressed at a high level in WT and higher in all Ox strains. In contrast, the *aroB* transcript levels, encoding 3-dehydroquinate synthase in 3-dehydroquinate (DHQ) synthesis, were lower in WT and Ox-A strains under both BG_11_ and BG_110_ + (NH_4_)_2_SO_4_ conditions. It is interesting to note that the *glgX* gene transcript kept track of the gene involved in the breakdown of glycogen. High levels of *glgX* transcript were expressed regularly in both WT and Ox strains, and the (NH_4_)_2_SO_4_ treatment only slightly lowered *glgX* transcript levels in Ox strains relative to WT. On the other hand, in carbon flux via the acetyl-CoA route, the *accA* gene transcript, encoding acetyl-CoA carboxylase subunit A, was highly accumulated during the late-log phase in all strains, except for the Ox-A strain, which had a lower amount of *accA* transcript under the (NH_4_)_2_SO_4_ condition. Additionally, under typical BG_11_ condition, the WT strain had higher level of *plsX* gene transcript expression than OX strains, which is connected to the synthesis of membrane lipids. The (NH_4_)_2_SO_4_ treatment had an influence on lower levels of *plsX* in all strains, while the highest amount was found in the Ox-A strain. Moreover, it is crucial to monitor membrane lipid degradation; high amounts of *lipA* transcript, encoding lipase A enzyme, were regularly expressed in all strains under normal BG_11_ condition, with Ox-A accumulating its lowest level. Unexpectedly, treatment with (NH_4_)_2_SO_4_ significantly caused an elevation of the *lipA* transcript level in the Ox-A strain (Fig. 2A,B). The recycling reaction of free fatty acid (FFA) products from membrane lipid degradation was also determined by *aas* transcript level, encoding acyl-ACP synthetase. Despite the Ox-A strain having the lowest level, both WT and Ox strains consistently accumulated a high level of *aas* transcript under both BG_11_ and BG_110_ + (NH_4_)_2_SO_4_ conditions. For another route from acetyl-CoA to PHB synthesis, the amount of *phaA* transcript engaged in PHB synthesis in Ox-B strain was expressed in a manner comparable to that of the WT, while lower levels of *phaA* transcript were found in the Ox-A and Ox-AB strains under the normal BG_11_ condition. The (NH_4_)_2_SO_4_ treatment resulted in an upregulated *phaA* transcript level in the Ox-A strain, which was consistent with a higher induction of PHB under the same condition.

### DPPH radical scavenging activity and components of mycosporine-like amino acids (MAAs) under ammonium sulphate supplementation

The DPPH radical scavenging capacity of methanol cell extracts from all strains was first assessed (Fig. 7A), and then pigment-free extracts were examined for DPPH radical scavenging activity and MAA component identified by HPLC (Fig. 7B,C). Results pointed out that late-log phase of growth had an impact on higher DPPH radical scavenging activity (73–78%) in cell extracts from all strains than log phase did (Fig. 7A). The (NH_4_)_2_SO_4_ treatment did not severely lower the ability of cell extracts to scavenge DPPH radicals. Although the pigment-free extracts had lower DPPH scavenging activity than methanol cell extracts by about 2–8 folds, it is worthwhile to note that at the late-log phase of cell growth, pigment-free extracts from Ox strains, which considerably included MAAs, had stronger DPPH scavenging activity than WT (Fig. 7B).

In Fig. 7C, the HPLC chromatogram, at both 334 and 310 nm, peak no. 4 (or P4) of methanolic extract at the retention time of 4.6 min was a major component of MAAs in WT under normal BG_11_ condition during late-log phase of cell growth, whereas P1, P2, P3, and P5 contributed only a minor fraction. Under typical BG_11_ condition, Ox strains, particularly Ox-A and Ox-AB, were shown to contain higher percentage of P4 and increased fraction of P1. Remarkably, all strains appeared to have the largest component of P4 exerted by the (NH_4_)_2_SO_4_ treatment, more than 80%, with the exception of the Ox-A strain detected by HPLC at 334 nm, which had the highest proportion of P1 (59%) instead. Furthermore, Ox-AB had a P2 component that had been inducibly increased by (NH_4_)_2_SO_4_ treatment up to 4%.

**Figure 8:**
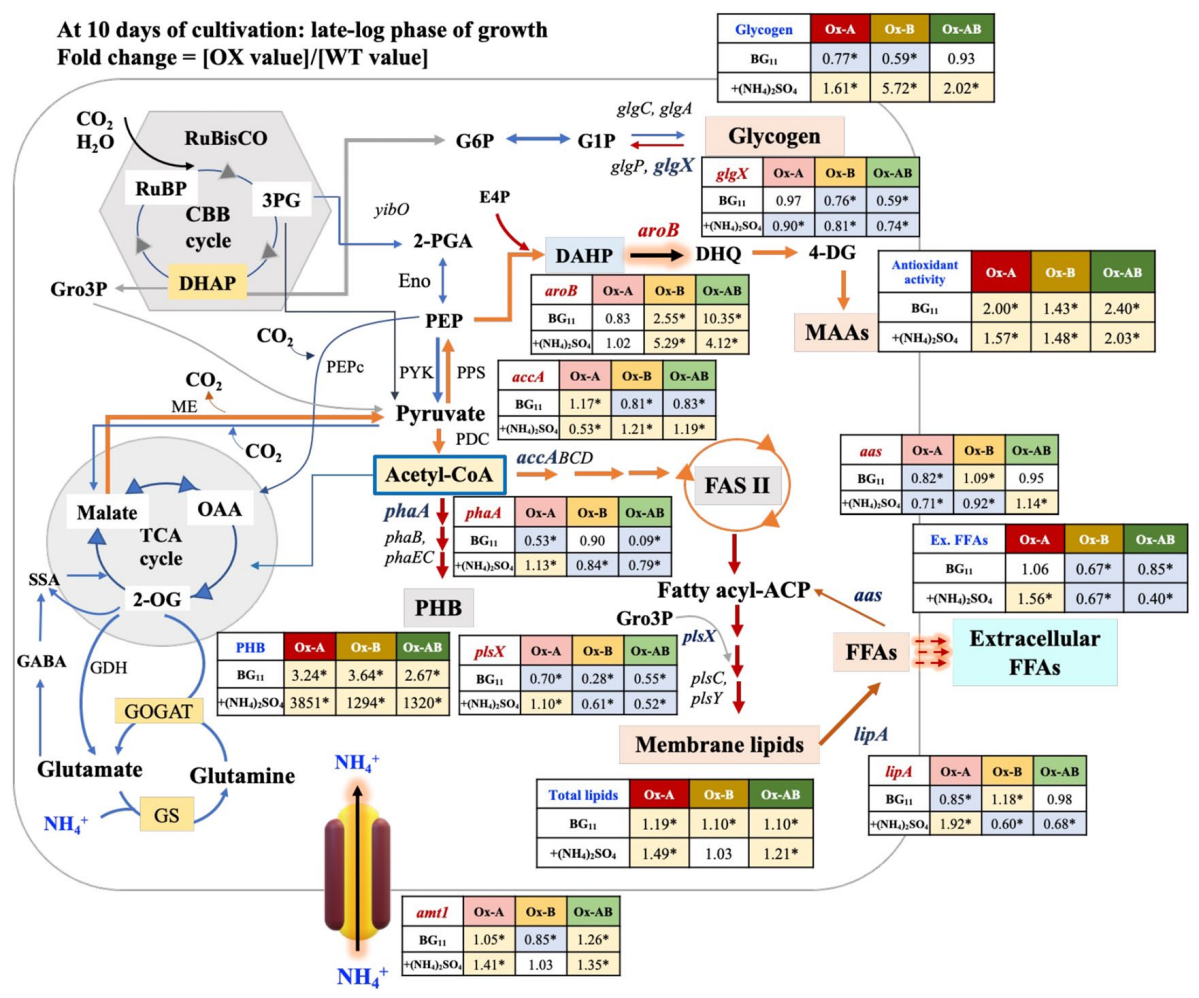
Summary of obtained results, including products and transcript amounts from three engineered (Ox) strains in comparison with those of *Synechocystis* sp. PCC 6803 wild type (WT) strain under normal BG_11_ and BG_110_ + (NH_4_)_2_SO_4_ conditions at late-log phase of growth (day 10). In each box, the number represents the fold change of that value of Ox strain divided by that value of WT under each condition. The statistical difference in the data between those values of WT and the Ox strain is shown by an asterisk at *P < 0.05. Outlined connection of nitrogen and carbon metabolism in cyanobacteria with the ammonium and nitrate transporters **(**modified from^1,16,17^). Pyruvate and acetyl-CoA are two key intermediates for many biosynthetic pathways, including glycogen, polyhydroxybutyrate (PHB), fatty acids and lipids, and amino acids and mycosporine-like amino acids. Metabolites, enzyme, and gene abbreviations: *amt1,amt2,* and *amt3*, encoding ammonium permeases; GABA, γ-aminobutyric acid; GABA-AT, γ-aminobutyrate aminotransferase; GDC, Glutamate decarboxylase; GDH, Glutamate dehydrogenase; GS, Glutamine synthetase; GOGAT, Glutamate synthase; OGDC, 2-oxoglutarate decarboxylase; SSA-DH, Succinic semialdehyde dehydrogenase; TCA cycle, Citric acid cycle; 2-OG, 2-Oxoglutarate; ME, Malic enzyme; OAA, Oxaloacetate; *pepc*, encoding PEP carboxylase; PEP, phosphoenolpyruvate; E4P**,** Erythrose-4-phosphate; DAHP, 3-deoxy-D-arabino-heptulosonate-7-phosphate; *aroB*, encoding 3-dehydroquinate synthase; DHQ, 3-dehydroquinate; 4-DG, 4-deoxygadusol; MAAs, mycosporine-like amino acids; *pykF,* encoding Pyruvate kinase (PK); *pps,* encoding phosphoenolpyruvate synthase (PPS); PDH, Pyruvate dehydrogenase; *phaA,* encoding Acetyl-CoA acetyltransferase (*PhaA*); *phaB,* encoding Acetoacetyl-CoA reductase (*PhaB*); *phaEC,* encoding the heterodimeric PHB synthase (*PhaEC*); PHB*,* polyhydroxybutyrate; *accABCD*, encoding a multi-subunit acetyl-CoA carboxylase gene; FASII, Fatty acid synthesis system II; Fatty acyl-ACP; fatty acyl-(acyl carrier protein); Gro3P, glycerol-3-phosphate; *plsX, plsY* and *plsC* encoding putative phosphate acyl-transferases; *lipA,* encoding a lipolytic enzyme Lipase A; FFAs, Free fatty acids; *aas,* encoding acyl-ACP synthetase (AAS); PPS; phosphoenolpyruvate synthase, CBB cycle, the Calvin–Benson–Bassham cycle; RuBP, ribulose-1,5-bisphosphate; RuBisCO, ribulose-1,5-bisphosphate carboxylase/oxygenase; DHAP, dihydroxyacetone phosphate; 3PG, 3-phosphoglycerate; *yibO,* encoding 2,3-Bisphosphoglycerate-independent phosphoglycerate mutase; 2-PGA, 2-phosphoglycerate; *eno*, encoding Enolase (Eno); Glycogen synthesis; G6P, glucose 6-phosphate; G1P, glucose 1-phosphate; *glgC* and *glgA,* encoding Glycogen synthase; *glgP,* encoding Glycogen phosphorylase; and *glgX,* encoding Glycogen isomerase.

The fold change of transcripts and products under the late-log phase of growth between OX and WT is presented in Fig. 8. Ammonium supplementation had an impact on the increased fold change of PHB accumulation in OX strains compared to WT, although the OX strain preferred to accumulate glycogen content rather than PHB (Figs. 6 and 8). When compared to WT, OX strains appeared to have a lower fold change in the *glgX* transcript level, which is implicated in the breakdown of glycogen. On the other hand, increased antioxidant activity was found in OX strains, with a higher fold change in comparison with WT.

## Discussion

In this study, we metabolically constructed the engineered *Synechocystis* sp. PCC 6803 strains by overexpressing *amt1* and *aroB* genes related to ammonium transporter (AMT) and 3-dehydroquinate (DHQ) synthesis, an upstream to aromatic amino acid and mycosporine-like amino acid syntheses, respectively. Three engineered strains, including the *amt1*- overexpressing strain (Ox-A), the *aroB*-overexpressing strain (Ox-B), and the double *amt1_aroB-*overexpressing strain (Ox-AB), had different metabolic flows exerted by ammonium sulphate ((NH_4_)_2_SO_4_) treatment compared with normal BG_11_ containing NaNO_3_ as a major nitrogen source^5,6,35^.

Lower growth rates of all strains were caused by (NH_4_)_2_SO_4_ supplementation when compared to normal BG_11_ condition (Fig. 3). According to previous findings, the PSII was found to be disrupted by the higher concentration of NH_4_^+^ in the medium, which increased the toxicity to the cell in cyanobacteria, algae, and plants^36–40^. Additionally, gene overexpression with interruption of the *psbA2* gene by double homologous recombination contributed to the reduced growth of the Ox strains, including Ox-A and Ox-B. Regarding ammonium sulfate supplementation, all *psbA* genes (*psbA1*, *psbA2*, *psbA3*) encoding D1 protein in the PSII were indicated to contribute to ammonium tolerance in *Synechocystis* sp. PCC 6803^41^. However, the Ox-A and Ox-AB strains with *amt1* gene overexpression grew better with higher growth rate than other strains (Fig. 3). We also demonstrated that the intracellular pigment contents of Ox strains, including chlorophyll* a* and carotenoids, were slightly different and lower than in WT while growing in normal BG_11_ containing NaNO_3_. However, the *aroB-*overexpressing strain (Ox-B) strain, which had the lowest pigment contents, was especially affected by the replacement of (NH_4_)_2_SO4 in the medium (Fig. 3C–F). We thoughtfully speculated that *aroB* overexpression might promote pyruvate conversion to PEP and DAHP, respectively, a crucial substrate for DHQ synthesis by DHQ synthetase (encoded by *aroB* gene) (Fig. 8). This would result in lower flow direction from pyruvate to carotenoid and chlorophyll biosynthesis. There was evident support that the Ox-B cells, growing in BG_110_ + (NH_4_)_2_SO_4_ during the first 4 days of cultivation (Fig. 4), showed an apparently light green culture in accordance with their lower OD_730_ and pigment contents.

For carbon flow-directed products, we showed that *Synechocystis* sp. PCC 6803 WT cells regularly utilized NaNO_3_ in typical BG_11_ growth medium for intracellular lipids and stored glycogen as a major carbon storage with a trace quantity of polyhydroxybutyrate (PHB) (Figs. 5 and 6). While WT cells maintained their intracellular lipid levels in the log and late-log phases of growth, they secreted more free fatty acids (FFAs) in the late-log phase. The *amt1* and/or *aroB* overexpression in *Synechocystis* sp. PCC 6803 showed a significant increase of intracellular lipid levels under normal BG_11_ condition (Fig. 5). Our results indicated that *amt1* overexpression in Ox-A, involved in ammonium transporter, dramatically increased the carbon flux required to synthesize intracellular lipids (29.6%w/DCW) and extracellular FFAs (17.5%w/DCW) under the influence of (NH_4_)_2_SO_4_ treatment, in particular, during the late-log phase of growth (Fig. 8). This finding was also supported by the increased fold changes of *plsX*, related to membrane lipid synthesis, and *lipA*, related in FFA production from membrane degradation, at transcript levels of about 1.13 and 1.92, respectively, when compared with those of WT. It is worth noting that when *lipA* transcript level was upregulated, this would indicate that intracellular FFA levels were also elevated to an excessive level, which could cause cell damage by disrupting the electron transport chain and destabilizing proteins located on thylakoid membranes^28,42–44^. Therefore, in order to lessen FFA toxicity, cells have the ability to recycle FFAs into fatty acyl-ACP or secret FFA out of cells. Additionally, the Ox-A strain favored the production of lipids while accumulating the least amount of glycogen in order to balance the metabolism and storage of carbon (Fig. 6). In contrast, the *aroB* overexpression in Ox-B considerably contributed to DHQ synthesis, preferred to retain a high amount of glycogen (5.72 times more than WT), and kept lipids levels steady under the (NH_4_)_2_SO_4_ condition (Figs. 6 and 8). It is in line with results of *glgX* transcript amounts related to glycogen breakdown, which demonstrated a lower level in Ox-B strain when compared to WT, with a 0.81 fold difference (Fig. 8). Moreover, we also demonstrated that at the late-log phase of growth, all Ox strains had more accumulations of PHB than the WT strain, albeit having less than 5%w/DCW. Despite the fact that nitrogen deprivation is known to cause a significant increase in PHB^45,46^, we suggested that ammonium treatment may have had a minor impact on the ability of the *amt1-*overexpressing strain (Ox-A) to produce more PHB during the late-log phase of growth.

On the other hand, we demonstrated that the component of mycosporine-like amino acids (MAAs) component of nitrogen flow-directed products was partially responsible for the DPPH radical scavenging activity (Fig. 7). Under ammonium treatment, methanol extracts from all Ox strains, especially Ox-B and Ox-AB strains, had a notable increase in DPPH radical scavenging activity. Results suggested that the *aroB* overexpression contributing to the synthesis of DHQ, a crucial intermediate in the biosynthetic pathways for aromatic amino acids and MAAs, increased the capacity of antioxidant activity. It is also crucial to take into account that the late-log growth stage significantly raised DPPH radical scavenging activity and caused a greater fold increase than WT (Fig. 8). On the other hand, by using the GS/GOGAT cycle of glutamate and glutamine synthesis, *amt1* overexpression, which encodes ammonium permease in the ammonium transporter, induced a certain improvement in the intracellular nitrogen pool^16,47^. According to previous studies, after 24–48 h of culture of *Synechocystis* cells under the NH_4_Cl condition, numerous amino acids were enhanced in comparison to the NaNO_3_ condition due to the different mechanism of nitrogen assimilation in the GS/GOGAT cycle between these two conditions^48,49^. In recent study, the *aroB* gene overexpression in *Escherichia coli* markedly enhanced aromatic amino acid (AAA) production^50^.

In cyanobacteria and algae, mycosporine-like amino acids (MAAs) have been predicted to synthesize via the first part of the shikimate pathway, where 3-dehydroquinate (DHQ) acts as a precursor for MAAs via gadusols^51,52^. MAAs in microalgae play a role in protective actions for survival against UV radiation, salinity, and other environmental challenges^53^. Mycosporine-glycine is the major type of MAAs, and it can be transformed into secondary MAAs such as shinorine, porphyra-334, palythine-serine, and others^52,54,55^. In the methanolic extract of the cyanobacterium *Anabaena doliolum*, three MAAs, including mycosporine-glycine, porphyra-334, and shinorine, were apparently identified by HPLC^56^. It is consistent with our HPLC at 334 nm results that demonstrated three distinct peaks of MAAs, including P4, P2, and P1, with retention times of 4.6, 2.9, and 2.1 min, respectively, and a small peak of P3 at a retention time at 3.9 min (Fig. 7). However, when detected by HPLC at 310 nm, the main P4 was dominant under both conditions (Fig. 7C). Prior research has confirmed that UV exposure caused the production of MAAs in *Synechocystis* sp. PCC6803, including mycosporine-taurine (M-tau)^31,57^, dehydroxxylusujirene^31,57^, M-343^57^, and mycosporine glycine^58^. The P1 to P5 fractions yielded similar findings to HPLC data of methanolic MAAs extract from a cyanobacterium *Anabaena doliolum*^56^, even though we did not identify each separated peak using HPLC with diode array detector. To pinpoint the exact MAA type, a particular extraction and detection approach for MAAs in *Synechocystis* sp. PCC6803 is required. Under typical BG_11_ condition during late-log phase of growth, the component of MAAs in *Synechocystis* sp. PCC 6803 WT strain consisted mostly of P4 fraction (52%),with a small portion of P1 (8%), P2 (1%), and P3 (1%) and a substantial proportion of P5 (38%). Our finding suggested that the mycosporine-glycine component, a primary component previously identified in *Anabaena doliolum*^56^, was present in a comparable HPLC chromatogram of the P4 fraction at 4.6 min retention time. Despite the fact that shinorine was defined as the MAAs component of the P1 fraction (rt = 2.1 min)^56^, the *Synechocystis* sp. PCC6803 wild type was previously reported to lack shinorine^59^. As in comparison with WT, the results indicated that the *amt1* and/or *aroB* overexpression had a substantial impact on the increased major component of P4 and P1. Furthermore, it was strikingly changed on the MAAs’ component induced by BG_110_ + (NH_4_)_2_SO_4_ medium. When ammonium was used as the nitrogen source, the P4 composition in the WT strain was dramatically boosted by up to 80%. A similar increase in P4 fraction was present in Ox-B and Ox-AB, with a certain composition of about 87% and 88%, respectively. In addition, the P2 component was apparently increased in Ox-AB by up to 4% in this condition, detected by HPLC at 334 nm. In contrast, the prominent component in Ox-A shone a high light on P1 fraction, up to 59%, as a result of ammonium treatment. Our finding thus revealed that the strategy by which *Synechocystis* sp. PCC 6803 utilized distinct nitrogen sources, in this case NaNO_3_ and (NH_4_)_2_SO_4_, had an impact on the component of MAAs that was connected to its antioxidant ability. Our findings demonstrated that Ox-AB, which had higher compositions of P4 and P2 than WT, had the best capacity for DPPH radical scavenging due to the combined overexpression of *amt1* and *aroB* genes. In addition, it was previously discovered that mycosporine-2-glycine (M2G), which is more active than other MAAs such as shinorine, porphyra-334, and palythine, efficiently increased anti-inflammatory and antioxidant properties by blocking the formation of advanced glycation end-products (AGEs) in lipopolysaccharide-stimulated RAW 264.7 macrophages^60^. Furthermore, increasing the nitrate and phosphate content in the medium proved another effective way to promote MAAs synthesis by *Fischerella* sp. F5^61^.

## Methods

### Strains and culture conditions

The host propagation, *Escherichia coli* DH5α strain was grown either on an agar plate or in a liquid medium of Luria Bertani (LB) containing 35 µg/mL of chloramphenicol (Cm) at 37 °C. Cyanobacterium *Synechocystis* sp. PCC 6803 wild type (WT) was derived from the Berkeley strain 6803 isolated from fresh water in California, USA^62^. *Synechocystis* sp. PCC 6803 strain was cultivated in normal BG_11_ medium^35^ using a rotary shaker at 28 °C and continuous light illumination of 50 µmol photons m^-2^ s^-1^. All engineered strains in this study, including Ox-A, Ox-B, and Ox-AB (Table 1) were cultured in a normal BG_11_ medium containing 35 µg/mL of chloramphenicol at the same growth condition.

### Constructions of recombinant plasmids

To construct the recombinant pECm_*amt1,* pECm_*aroB,* and pECm_*amt1/aroB* plasmids (Table 1), the pEERM vector containing the the chloramphenicol resistance cassette gene (*Cm*^*r*^) was used as a model vector for cloning and expressing genes^63^. The sequences of *amt1 (sll0108)* and *aroB (slr2130)* genes were retrieved from the Cyanobase database. The *amt1* (*sll0108*) and *aroB* (*slr2130*) gene fragments with sizes of 1548 and 1398 bp were amplified by PCR using a pair of sll0108_F and sll0108_R primers, and another pair of slr2130_F and slr2130_R primers, respectively (Supplementary Information Table S1). The recombinant pECm_*amt1* plasmid was constructed by inserting a homologous *amt1* gene fragment into the pEERM vector between the *XbaI* and *BcuI* restriction sites. Additionally, the recombinant pECm_*aroB* plasmid was created by inserting an *aroB* gene fragment into the pEERM vector between the *BcuI* and *PstI* restriction sites. Ultimately, the recombinant pECm_*amt1/aroB* plasmid was constructed by introducing the *aroB* fragment into the recombinant pECm_*amtI* plasmid between the *BcuI* and *PstI* restriction sites.

### Natural transformation of recombinant plasmids into *Synechocystis* sp. PCC 6803 cells

The host *Synechocystis* sp. PCC 6803 wild type (WT) cells were grown in a normal BG_11_ medium until the optical density was about 0.3–0.5. Cell culture (50 mL) was harvested by centrifugation at 5000 rpm (2516*×g*) for 10 min. The obtained cell pellets were washed with fresh BG_11_ medium and harvested again by centrifugation at 5000 rpm (2516*×g*) for 10 min. Those cell pellets were condensed in 0.5 mL of new BG_11_ medium. After that, 1 µg of each recombinant plasmid was separately added to condensed WT cells and incubated at 28 °C for 6 h by inverting the tubes every 2 h. Then, the sample mixture was spread on a 0.45 µm sterile nitrocellulose membrane placed over a normal BG_11_ agar plate overnight. Next, that membrane was transferred to new BG_11_ agar plate containing 35 µg/mL chloramphenicol. After several weeks of incubation, the survival colonies were collected and examined for their gene location and segregation by PCR analysis using specific pairs of primers (Supplementary Information, Tables S1 and S2).

### Cell cultivation and ammonium sulfate treatment

Initially, cell stock cultures with a mid-log phase of growth were harvested by centrifugation at 6000 rpm (3622*×g*) for 10 min and transferred into normal BG_11_ medium containing 17.6 mM NaNO_3_, and BG_110_ medium (without NaNO_3_) containing 8.8 mM (NH_4_)_2_SO_4_ (BG_110_ + (NH_4_)_2_SO_4_). The initial OD_730_ of cultivation was approximately 0.1 and continuously cultured for 16 days.

### Determinations of cell growth and pigment contents

*Synechocystis* cell growth was monitored by a spectrophotometer during cultivation. The pigment contents, including chlorophyll *a* (chl *a*) and carotenoids, were extracted and determined as described in^64,65^. One milliliter of cell culture was harvested and centrifuged at 6000 rpm (3622*×g*) for 10 min. To extract the pigments, 1 mL of *N*,*N*-dimethylformamide (DMF) was mixed with cell pellets. After quickly spinning, the supernatant of extracted pigments was measured for absorbance (Abs) at 461, 625, and 664 nm using a spectrophotometer. The data were later calculated and normalized to cell numbers corresponding to 10^8^ of the cells.

### Measurement of oxygen evolution rate

Five mL of cell culture were centrifuged at 6000 rpm (3622*×g*) for 10 min. Cell pellets were resuspended with 2 mL of fresh BG_11_ medium and incubated in the darkness for 30 min. After that, that cell suspension was measured for oxygen evolution by a Clark-type oxygen electrode (Hansatech instruments Ltd., King’s Lynn, UK) at room temperature (25 °C). The unit of the O_2_ evolution rate was presented as µmol/mg chlorophyll *a*/h.

### Reverse transcription polymerase chain reaction

Fifteen mL of cell culture was harvested by centrifugation at 6000 rpm (3622*×g*), 10 min, and the total RNAs was extracted by using 1 mL of TRIzol^®^ Reagent (Invitrogen, Life Technologies Corporation, Carlsbad, CA, USA). The isolated RNAs were treated with RNaseI-free DNAseI (Fermentas, Carlsbad, CA, USA) to remove the DNA contamination before converting them to cDNA using ReverTra Ace^®^ qPCR RT Master Mix (TOYOBO Co., Ltd., Osaka, Japan). Then, the cDNA product was used as a template for PCR analysis of interest genes, including *amt1, aroB, glgX, phaA, accA, aas, plsX, lipA,* with *16s* rRNA as a reference*.* The RT-PCR primers were listed in Supplementary Information Table S1. The PCR conditions, using KOD polymerase, were the initial denaturation at 98 °C for 3 min, followed by proper cycles of each gene at 98 °C for 10 s, the primer melting temperature (Tm) for 10 s, 68 °C for 10 s to extend the DNA strand, and 68 °C for 5 min at the last step. The cycle number and Tm of each primer pair were shown in Supplementary Information Table S1. PCR products were verified by electrophoresis on 1.0% (w/v) agarose gels and the intensity of bands was determined by using a Syngene Gel Documentation (SYNGENE, Frederick, MD, USA).

### DPPH radical scavenging assay

Ten mL of cell culture was harvested by centrifugation at 6000 rpm (3622*×g*) for 10 min. One mL of absolute methanol was mixed with cell pellets and vortexed, then incubated this mixture solution at 4 °C overnight. The supernatant was collected by centrifugation at 12,000 rpm (8050*×g*), for 10 min. Then, sample solution (500 µL) was taken into a new microtube and filtrated through 0.2 µm porous syringe filter, then 500 µL of 0.1 mM 2,2-diphenyl-1*-*picrylhydrazyl *(*DPPH*)* prepared with methanol dissolution was added and vortexed to mix the reaction^66^. After 10 min in the dark, the absorbance at 517 nm was measured with a spectrophotometer. Finally, the calculation of the percentage of radical scavenging activity (%) was obtained by this equation; [(A_control_ − A_sample_)/A_control_] × 100, A = absorbance at 517 nm.

### Extraction of mycosporine-like amino acids (MAAs) and HPLC detection

The MAAs extraction and the HPLC detection method were modified from^56,67^. Thirty mL of cell culture was harvested by centrifugation at 6000 rpm (3622*×g*) for 10 min. To extract MAAs, 1 mL of absolute methanol HPLC grade was mixed with the cell pellets, vortexed, and incubated overnight at 4 °C (Supplementary information Fig. S3). The supernatant was collected by centrifugation at 10,000 rpm (3,622 × *g*), for 10 min and later dried by vacuum evaporation at 45 °C for 4 h. Distilled water (500 µL) was added to redissolve the extracted products and then removed pigments by adding 200 µL of CHCl_3_ (Supplementary information Fig. S4). After vortexing for a few minutes and centrifuged at 10,000 rpm (3622*×g*) for 10 min, the organic and aqueous phases were completely separated. Finally, the aqueous phase was filtered through Whatman Nylon filter media with a polypropylene housing 0.2 microns, 13 mm, and collected in a glass vial for HPLC analysis (Shimadzu HPLC LGE System, Kyoto, Japan). A reverse phase column (Inertsil ODS-3, 4.6 mm × 250 mm; GL Sciences Inc., Tokyo, Japan) was used and performed with a flow rate of 1.0 mL/min. The running buffer was 0.02% (v/v) acetic acid in ultrapure water (UP). The MAA compositions were calculated from the HPLC peak area of each retention time (rt), as identified according to^56,67^, including P1 (rt = 2.1), P2 (rt = 2.9), P3 (rt = 3.9), P4 (rt = 4.6), and P5 (rt = 9.6). The HPLC chromatogram was depicted in Supplementary Information, Figs. S5 and S6.

### Extraction of intracellular lipids and extracellular free fatty acids (FFAs)

Ten mL of cell culture was harvested by centrifugation at 6000 rpm (3622*×g*) for 10 min. Intracellular lipids were extracted from the harvested cell pellets, while the extracellular FFAs were extracted from the culture medium according to the method of^68^ with a slight modification. The solvent mixture (1 mL) of chloroform (CHCl_3_): methanol (CH_3_OH) with a ratio of 2:1 was added into a glass tube of cell pellets and incubated in a water bath at 37 °C for 2 h. Then, 500 µL of 0.88% (v/v) potassium chloride (KCl) was added and vortexed for a few seconds. After centrifugation of the reaction mixture tube at 3000 rpm (906*×g*) for 5 min, the lower organic phase containing lipids was collected. Then, the chloroform solvent was evaporated at 70 °C.

### Determination of total lipid and extracellular FFA content

Either the total lipid or extracellular FFA content was determined by the potassium dichromate oxidation reaction method^69^. A solution of K_2_Cr_2_O_7_ (0.18 M, 0.5 mL) and conc. sulfuric acid were added to the glass tube of extracted lipids. The reaction mixture was boiled at 105 °C for 30 min. After cooling it down to room temperature, distilled water (0.5 mL) was added into the reaction sample before measuring its absorbance at 600 nm (A_600_) using a spectrophotometer. In this experiment, canola oil was used as a commercial standard and prepared as same as the sample. The unit of lipid or FFA content was the weight percentage of dry cell weight (%w/DCW).

### Glycogen extraction and determination of glycogen content

Glycogen was extracted by alkaline hydrolysis (modified from^70^). Five mL of cell culture was harvested by centrifugation at 6000 rpm (3622*×g*), for 10 min. Cell pellets were collected, and mixed with 600 µL of 30% (v/v) KOH. The mixture was then heated at 90 °C for 1 h. The supernatant was separated by centrifugation at 12,000 rpm (14,489*×g*) for 10 min, then it was transferred into a 1.5 mL microcentrifuge tube. After adding 900 mL of absolute ethanol into the solution tube, it was incubated at − 20 °C for overnight to precipitate glycogen. The glycogen sediment fraction was harvested by centrifugation at 12,000 rpm (14,489*×g*) 4 °C for 10 min, and completely dried at 60 °C for overnight. After that, the sediment was dissolved with one mL of 10% (v/v) H_2_SO_4_. To determine glycogen content, the dissolved sample (0.5 mL) was taken to mix with 1 mL of anthrone solution (2 g/L anthrone dissolved in concentrated H_2_SO_4_). The reaction mixture was vigorously vortexed and subsequently heated at 90 °C for 10 min. The sample solution was then measured by a spectrophotometer at the absorbance of 625 nm. The commercial glycogen standard was prepared as same as the sample. In this study, the unit of glycogen content was the percentage of glycogen per dried cell weight (%w/DCW).

### Determination of PHB content by HPLC instrument

Five mL of cell culture were harvested by centrifugation at 6000 rpm (3622*×g*), for 10 min. One hundred µL of adipic acid (20 mg/mL) and 800 µL of concentrated H_2_SO_4_ were added into the tube of cell pellets and further boiled at 100 °C for 1 h for converting PHB to crotonic acid (modified from^71^). After that, 50 µL of the reaction mixture was diluted with 1.20 mL of ultrapure water. Then, one mL of solution was filtered through a PP Syringe filter 0.45 microns, 13 mm and collected in a glass vial for HPLC analysis (Shimadzu HPLC LGE System, Kyoto, Japan). A carbon-18 column with inert sustain 3 µm (GL-Sciences, Tokyo, Japan) was used and performed with a flow rate of 1.0 mL/min. The running buffer was 30% (v/v) acetonitrile in 10 mM KH_2_PO_4_ at pH 2.3. The amount of crotonic acid was detected at 210 nm of the UV detector. The commercial standard of crotonic acid was prepared as same as the samples. PHB content is expressed as a percentage of PHB per dried cell weight (%w/DCW).

### Supplementary Information

Supplementary Information.

## Data Availability

The data that support the findings of this study are available within the article and its supplementary files or from the corresponding author upon reasonable request.
