# Supplementary material for: Improved lipid production and component of mycosporine-like amino acids by co-overexpression of amt1 and aroB genes in Synechocystis sp. PCC6803
Source: Sci Rep. 2023 Nov 9;13:19439. doi: 10.1038/s41598-023-46290-x (PMC10636201; doi:10.1038/s41598-023-46290-x)

Supplementary Information

Table S1. Primers for gene amplification, confirmation of gene segregation, and determination of gene transcription were used in this study.

Name	Sequence (5' to 3')	Purpose of primer	Expected size (bp)	Cycles/Tm	Reference
Sll0108_F	TAGAGATCTAGAATGTCTAATT CGATATTGTC	PCR for amt1	1,548	30 /59.0 °C	This study
Sll0108_R	TAGAGAACTAGTTTATTCAGGG ACAGTGGCAC	PCR for amt1			This study
Slr2130_F	TAGAGAACTAGTTCAGTTAACC TGAAGATGGG	PCR for aroB	1,398	30 /60.0 °C	This study
Slr2130_R	TAGAGACTGCAGTTAGCCTAGG GTTGCCTTAA	PCR for aroB			
UUSsll0108_F	CACTATCACAGAGCTGGCTTA	Colony PCR			This study
UUSpsbA2_F	CACTCAGATAGGAGCCATCTTG C	Colony PCR			Eungrasamee et al. 2020
DDSpsbA2_R	CCCGTAGTTGTTCAATGATGAT GAT	Colony PCR			Eungrasamee et al. 2020
Cm_F	CGAGTTGATCGGGCACGTAA	Colony PCR			Eungrasamee et al. 2020
Cm_R	CAGCTCGAGGCTTGGATTCT	Colony PCR			Eungrasamee et al. 2020
pEbb_F	CATTACGCTGACTTGACGGG	Colony PCR			Eungrasamee et al. 2019
pEbb_R	AGGTATGTAGGCGGTGCTAC	Colony PCR			Eungrasamee et al. 2019
RTamtI_F480	TCCTTGATGTTCGGCAGCAG	RT-PCR for amt1	480	28 /56.0 °C (BG_{11})	This study
RTamtI_R480	CCAGCCGATCCAGAGAATTA	RT-PCR for amt1		29 /56.0 °C (BG_0+$(NH_4)_2SO_4$)	
RTaroB_F460	TCACTCCGTATTTAATTACCTCC GCC	RT-PCR for aroB	460	28 /52.0 °C (BG_{11})	This study
RTaroB_R460	ATGGTGGTGTCCAATCCGGA	RT-PCR for aroB		30 /52.0 °C (BG_{11}-N)	
RTphaA_F420	TCAGCCGGATAGAAT TGGACGAAGT	RT-PCR for phaA	420	28 /52.0 °C (BG_{11})	Eungrasamee et al. 2019
RTphaA_R420	CAAACAAGTCAAAATCTGCCA GGGTT	RT-PCR for phaA		30 /52.0°C (BG_0+$(NH_4)_2SO_4$)	
RTaccA_F428	ATGCACGGCGATCGAGGAGGT	RT-PCR for accA	428	32 /55.0 °C (BG_{11})	Eungrasamee et al. 2019
RTaccA_R428	TGGAGTAGCCACGGTGTACAC	RT-PCR for accA		30/55.0 °C (BG_0+$(NH_4)_2SO_4$)	
RTglgX_F360	GAGCTTCATCGAGGACGGAA	RT-PCR for glgX	360	32 /52.0 °C (BG_{11})	Eungrasamee et al. 2022
RTglgX_R360	GCCCGAATTGGGGTTGCGGG	RT-PCR for glgX		30 /52.0 °C (BG_0+$(NH_4)_2SO_4$)	
RTplsX_F488	AAGGGGTGGTGGAAATGGAA	RT-PCR for PlsX	488	35 /55.0 °C (BG_{11})	Towijit et al. 2018
RTplsX_R488	AAGTAGGTCCCTTCCTTCGG	RT-PCR for PlsX		35 /55.0°C (BG_0+$(NH_4)_2SO_4$)	
RTlipA_F379	TTGGCGGAGCAAGTGAAGCAAT	RT-PCR for lipA	379	30 /55.0 °C (BG_{11})	Eungrasamee et al. 2019
RTlipA_R379	CATGGACCAGCACAGGCAAAAT	RT-PCR for lipA		30 /55.0 °C (BG_0+$(NH_4)_2SO_4$)	
RTaas_F307	GTGGTTTATCGCCGATCAAG	RT-PCR for aas	307	31 /55.0 °C (BG_{11})	Eungrasamee et al. 2019
RTaas_R307	TTCCTGGCGGGGAACGGGAG	RT-PCR for aas		30 /55.0 °C (BG_0+$(NH_4)_2SO_4$)	
RT16SrRNA_F521	AGTTCTGACGGTACCTGATGA	RT-PCR for 16s	521	20 /56.0 °C (BG_{11})	Eungrasamee et al. 2019
RT16SrRNA_R521	GTCAAGCCTTGGTAAGGTTAT	RT-PCR for 16s		19 /56.0 °C (BG_0+$(NH_4)_2SO_4$)	

Table S2. PCR conditions for checking transformants

**Strains**	**Pair of primers**	**Cycles/Tm**	**Expected size (bp)**
Ox-A	UUSpsbA2/ DDSpsbA2	30 /57.0 °C	4,008
Ox-B	UUSpsbA2/ DDSpsbA2 for Ox-B	30 /57.0 °C	3,858
Ox-AB	UUSsll0108_F/Cm_R	30 /54.0 °C	4,028
	Cm_F/ pEbb_R		1,764
	pEbb_F/Sll0108_R	30 /56.0 °C	3,532
	Slr2130_F/pEbb_R	30 /56.0 °C	3,197

Figure S1 The original image of 1 %agarose gel electrophoresis. The transcript level of genes in **Figure 1B-D** and **Figure 2A** (including *amt1, aroB, phaA, accA, glgX, plsX, lipA, aas,* and *16s* rRNA in WT (lane no.2), Ox-A (lane no.3), Ox-B (lane no.4) Ox-ABC (lane no.5)) cultured under normal BG_{11} medium. Gels were analyzed by Syngene Gel Documentation instrument (SYNGENE, Frederick, MD, USA).

Figure 1B The native *psbA2* site of Ox-A strain

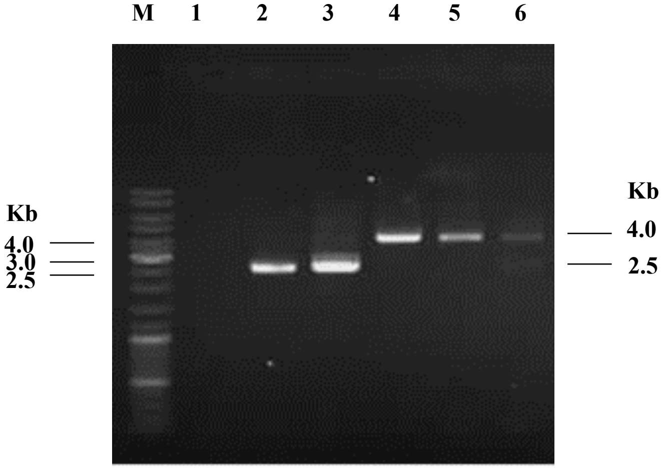

Figure 1C The native *psbA2* site of Ox-B strain

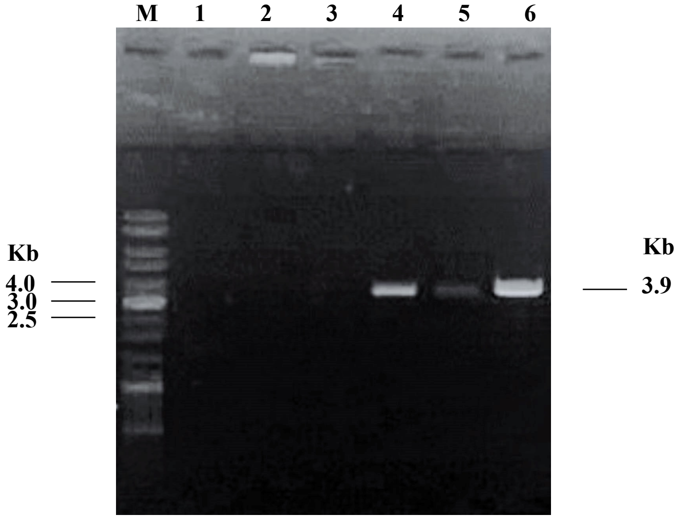

Figure 1D The native *psbA2* site of Ox-ABC strain

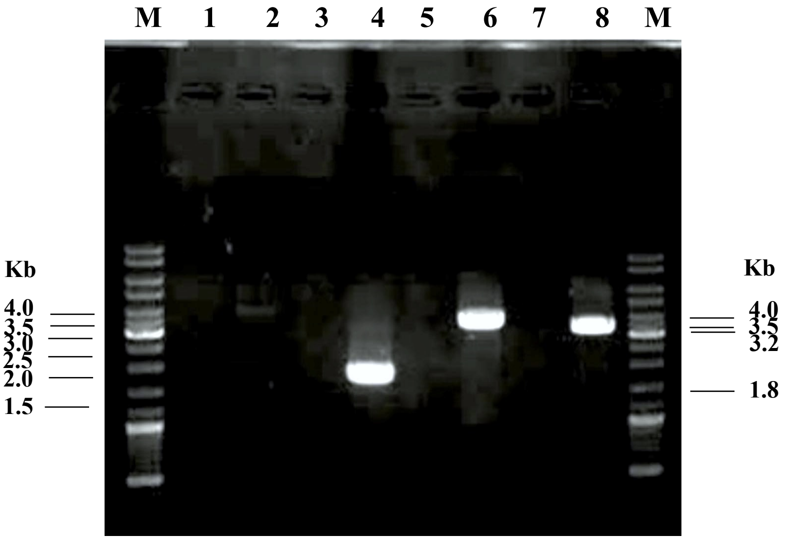

Figure S1 (continued) Original images of agarose gel electrophoresis from RT-PCR

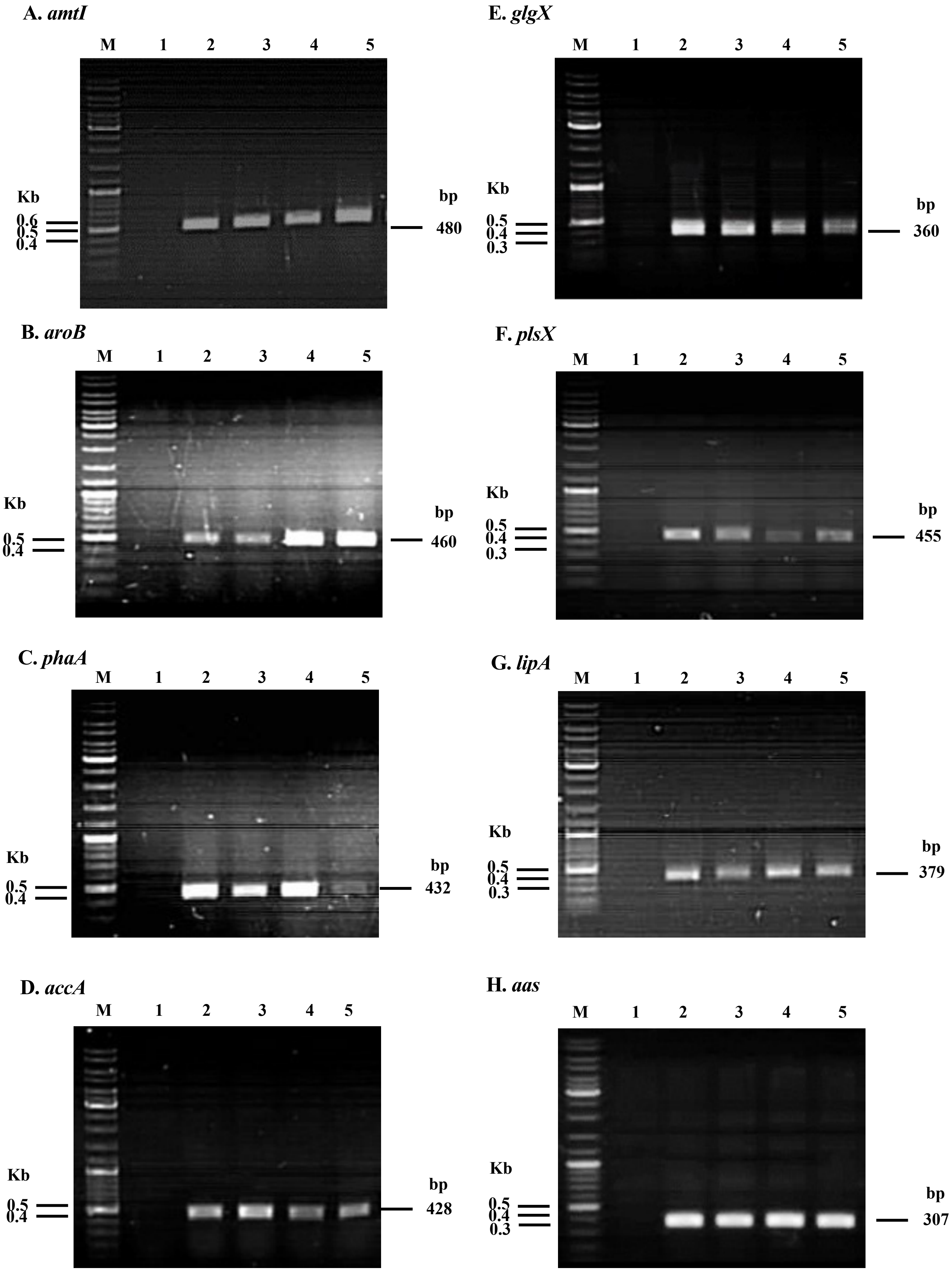

Figure S1 (continued) Original images of agarose gel electrophoresis from RT-PCR

I. ***16s rRNA***

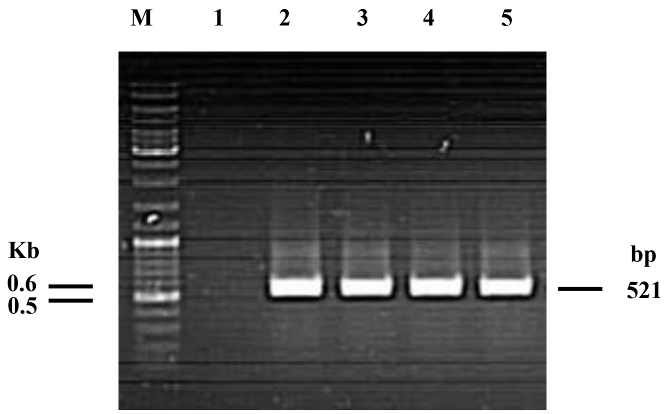

Note: all images are agarose gel electrophoresis, captured and analyzed by Syngene Gel Documentation instrument (SYNGENE, Frederick, MD, USA) with the 300 dpi resolution.

For the length of gels, we took the image of the agarose gel of PCR products visualized through the gel documentation instrument and captured the image length by bordering the concise area of those bands of DNA marker and the focused products.

Figure S2 The original image of 1 %agarose gel electrophoresis. The transcript level of genes in **Figure 2A,** including *amt1, aroB, phaA, accA, glgX, plsX, lipA, aas,* and *16s* rRNA in WT (lane no.2), Ox-A (lane no.3), Ox-B (lane no.4) Ox-ABC (lane no.5) cultured in BG_{11o}+$(NH_4)_2SO_4$ medium, respectively,

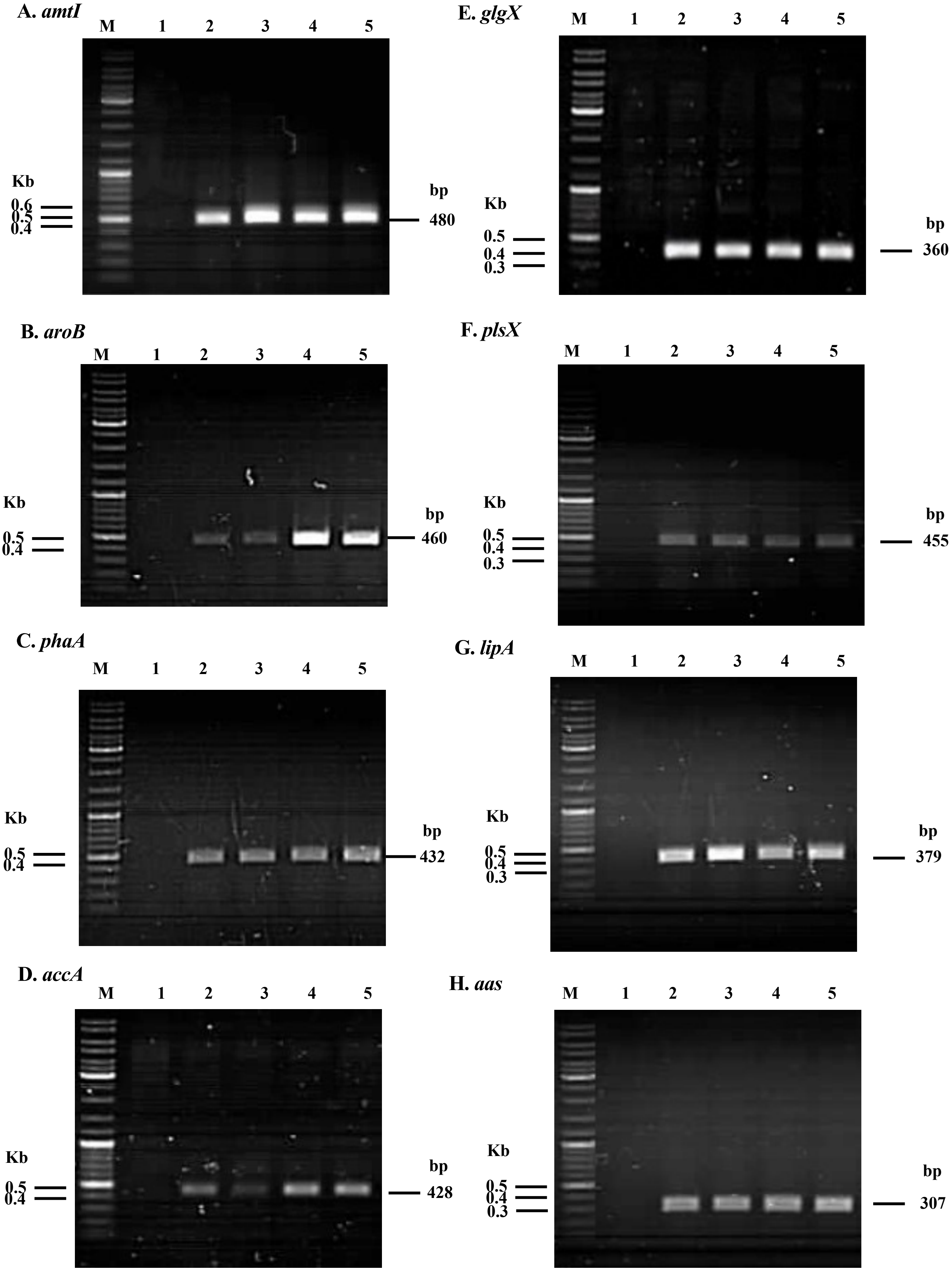

Figure S2 (continued) Original images of agarose gel electrophoresis from RT-PCR

I. *16s rRNA*

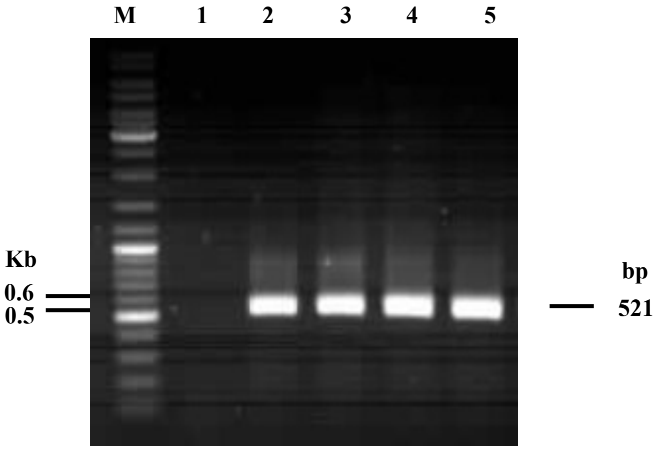

Note: all images are agarose gel electrophoresis, captured and analyzed by Syngene Gel Documentation instrument (SYNGENE, Frederick, MD, USA) with the 300 dpi resolution.

For the length of gels, we took the image of the agarose gel of PCR products visualized through the gel documentation instrument and captured the image length by bordering the concise area of those bands of DNA marker and the focused products.

Figure S3 Spectra of methanolic extract with pigments. Cell samples at the late-log (LL) phase of growth, both 10 mL and 5 mL cell cultures, were collected and extracted by absolute MeOH. The wavelength of methanolic extract was scanned between 220 and 750 nm by DS-C Cuvette Spectrophotometer (DeNovix®, USA).

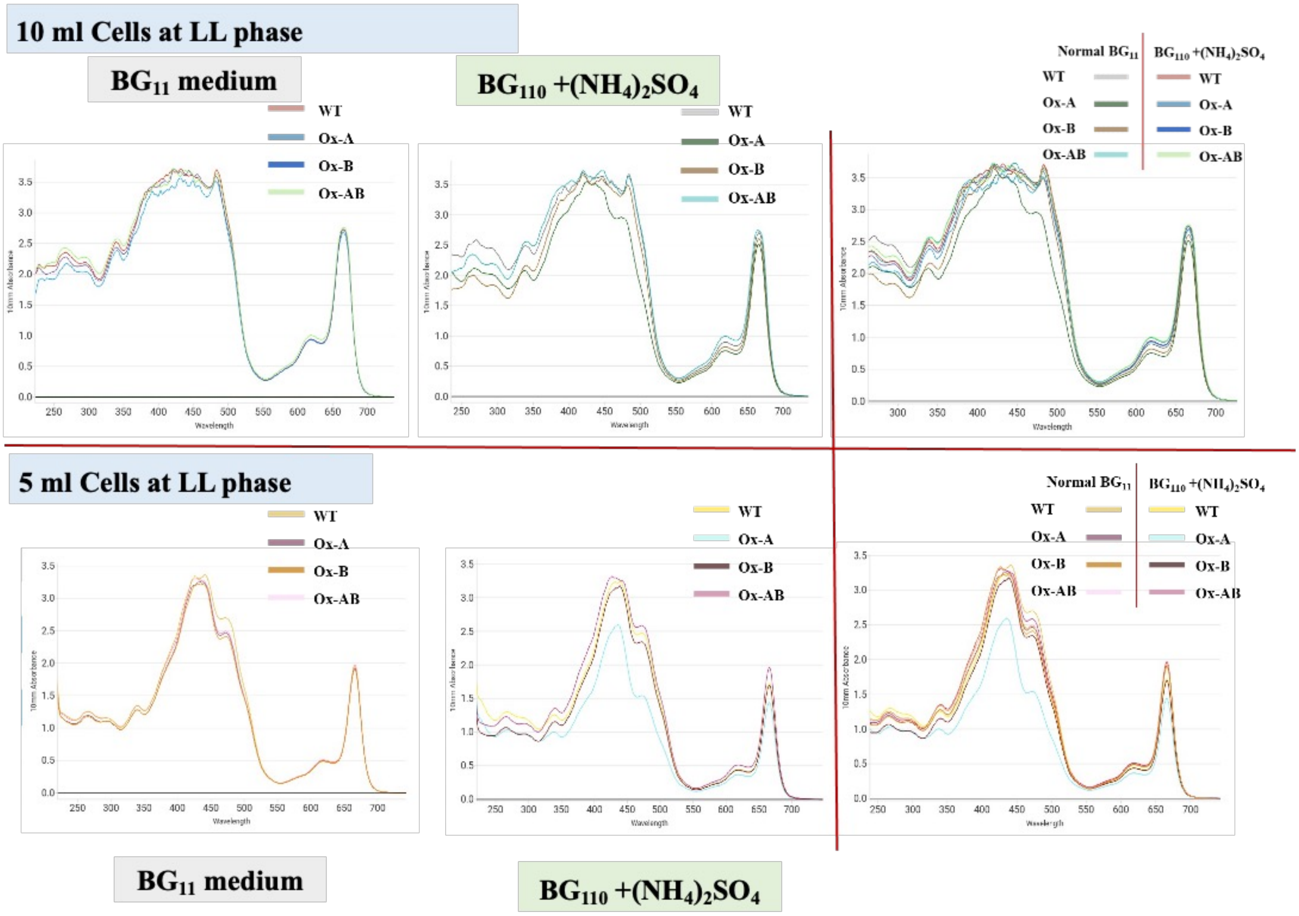

Figure S4 Spectra of methanolic extract without pigments. The pigments were removed by chloroform extraction. The extract solution was scanned for absorbances between 220 and 750 nm by DS-C Cuvette Spectrophotometer (DeNovix®, USA).

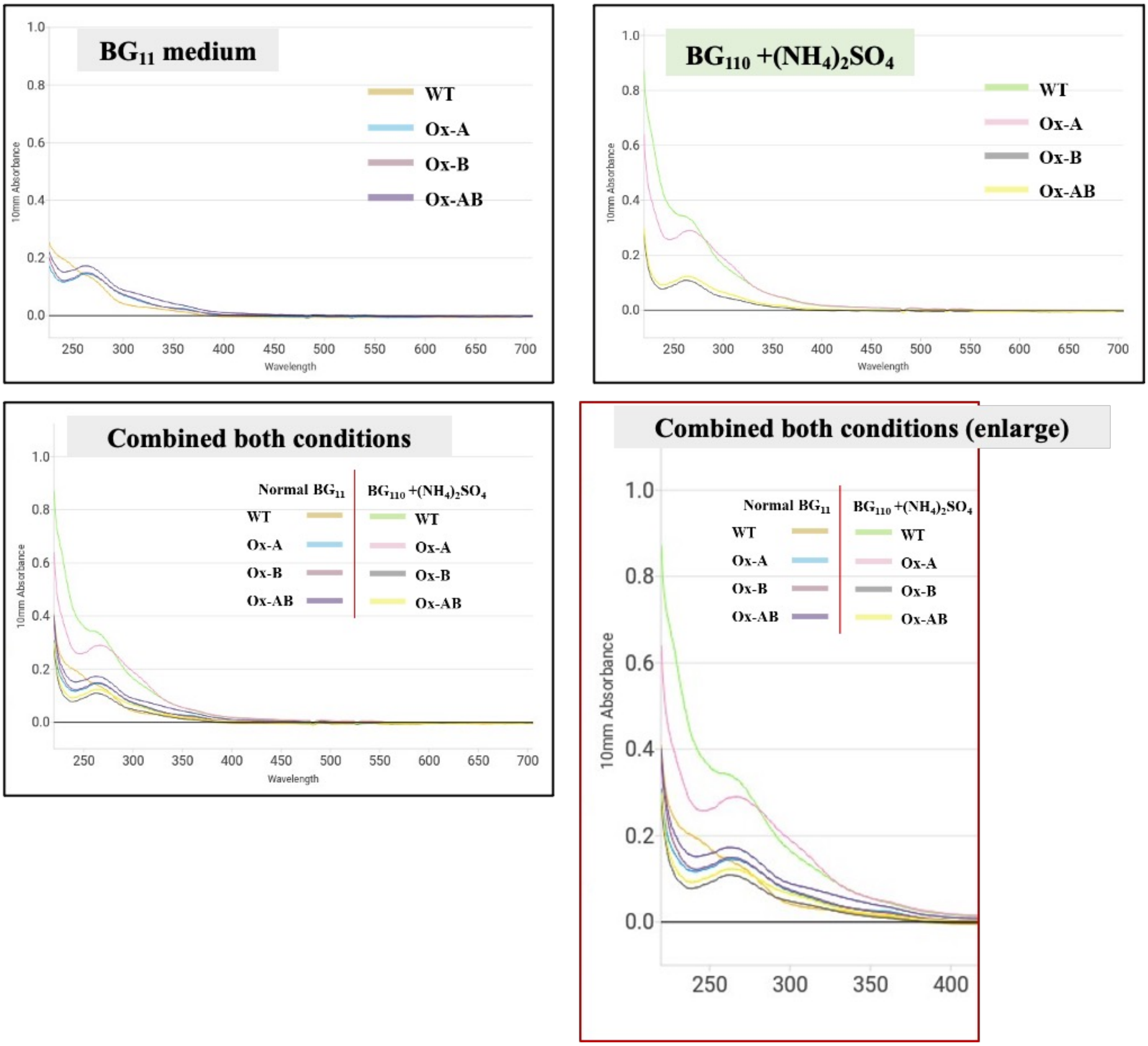

Figure S5 The HPLC chromatogram under normal BG_{11} condition : detection of MAAs at 334 and 310 nm. Methanolic extracts from cells at late-log phase of growth were used for MAAs analysis. (1) Peak 1 (P1, rt = 2.1), (2) Peak 2 (P2, rt =2.9), (3) Peak 3 (P3, rt = 3.9), (4) Peak 4 (P4, rt = 4.6), and (5) Peak 5 (P5, rt = 9.6).

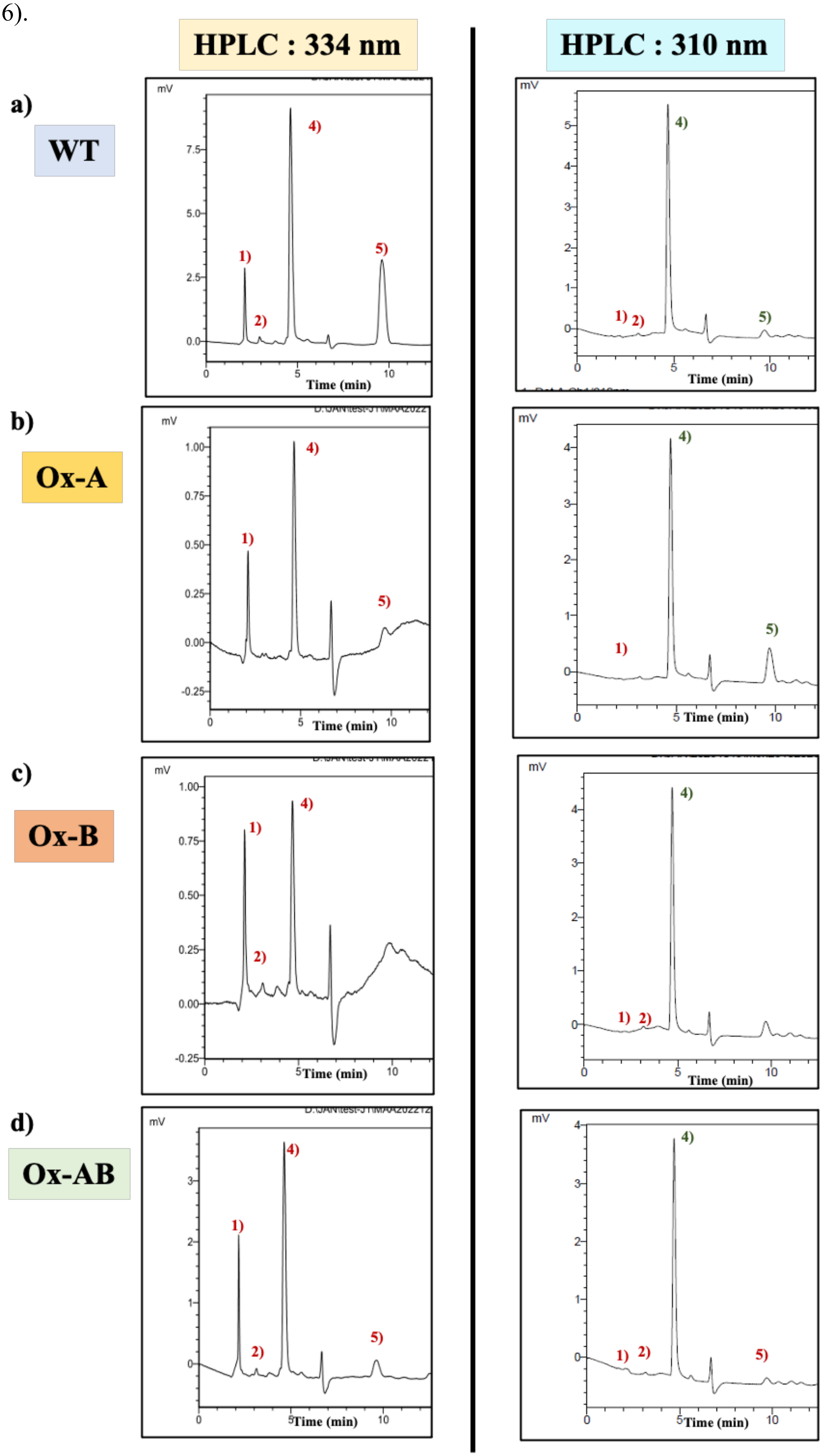

Figure S6 The HPLC chromatogram under BG_{11o}+$(NH_4)_2SO_4$ condition : detection of MAAs at 334 and 310 nm. Methanolic extracts from cells at late-log phase of growth were used for MAAs analysis. (1) Peak 1 (P1, rt = 2.1), (2) Peak 2 (P2, rt =2.9), (3) Peak 3 (P3, rt = 3.9), (4) Peak 4 (P4, rt = 4.6), and (5) Peak 5 (P5, rt = 9.6).

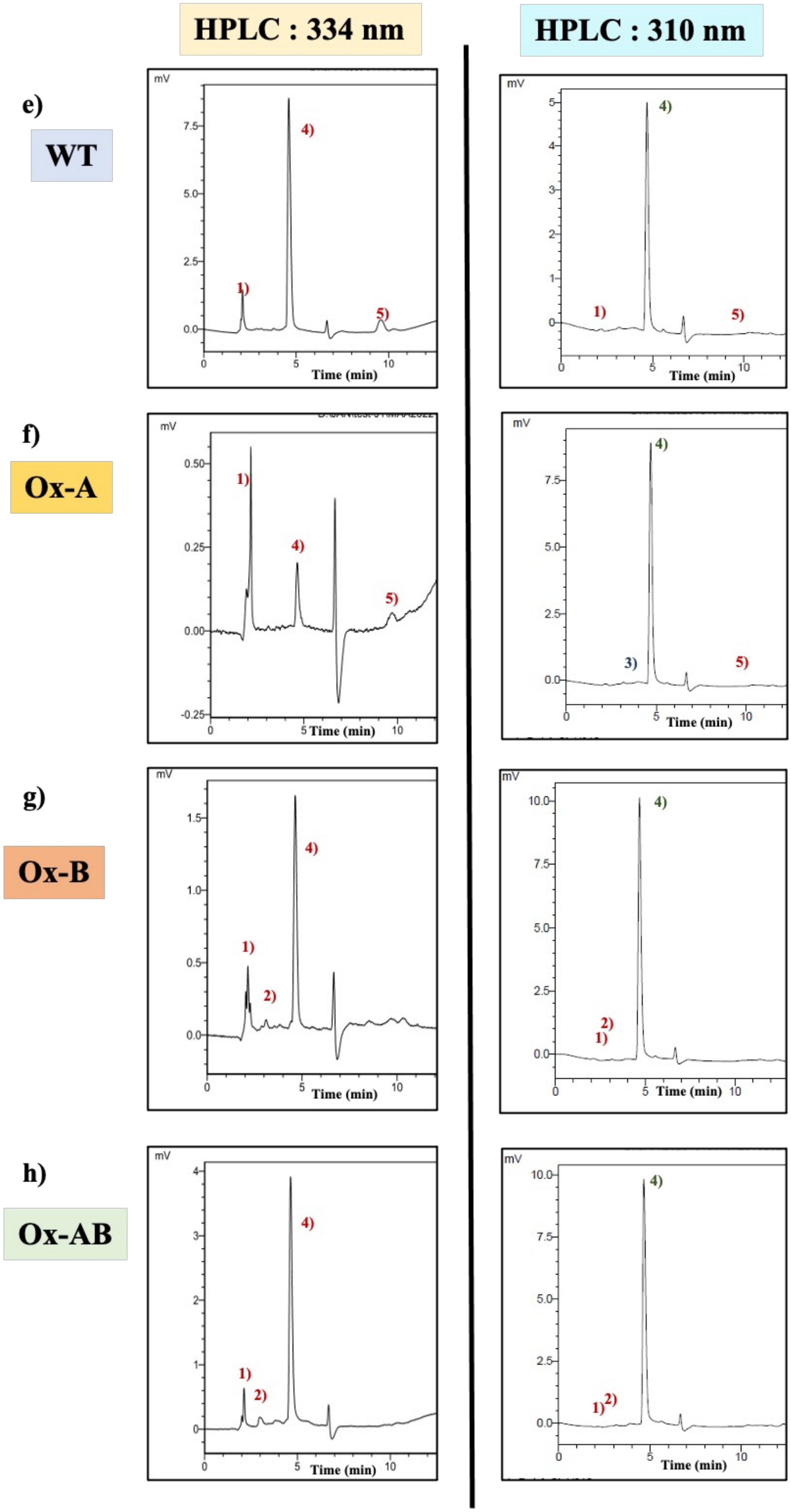

Supplement: Supplementary file 1 — Supplementary Information. [file 41598_2023_46290_MOESM1_ESM.pdf]
